# Exercise-induced IL-15 acted as a positive prognostic implication and tumor-suppressed role in pan-cancer

**DOI:** 10.3389/fphar.2022.1053137

**Published:** 2022-11-17

**Authors:** Zhiwen Luo, Zhong He, Haocheng Qin, Yisheng Chen, Beijie Qi, Jinrong Lin, Yaying Sun, Junming Sun, Xiaoping Su, Ziwen Long, Shiyi Chen

**Affiliations:** ^1^ Department of Sports Medicine, Huashan Hospital, Fudan University, Shanghai, China; ^2^ Department of Rehabilitation, Huashan Hospital, Fudan University, Shanghai, China; ^3^ Department of Orthopedics, Shanghai Pudong Hospital, Fudan University, Shanghai, China; ^4^ Laboratory Animal Center, Guangxi Medical University, Nanning, China; ^5^ Guangxi Key Laboratory of Oral and Maxillofacial Rehabilitation and Reconstruction, College & Hospital of Stomatology, Guangxi Medical University, Nanning, China; ^6^ Department of Gastric Cancer Sugery, Fudan University Shanghai Cancer Center, Shanghai, China

**Keywords:** pan-cancer, IL-15, prognosis, ferroptosis/cuproptosis, immune, exercise, multi-analyses

## Abstract

**Objective:** Exercise can produce a large number of cytokines that may benefit cancer patients, including Interleukin 15 (IL-15). IL-15 is a cytokine that has multiple functions in regulating the adaptive and innate immune systems and tumorigenesis of lung and breast cancers. However, the roles of IL-15 in other types of cancer remain unknown. In this article, we try to systematically analyze if IL-15 is a potential molecular biomarker for predicting patient prognosis in pan-cancer and its connection with anti-cancer effects of exercise.

**Methods:** The expression of IL-15 was detected by The Cancer Genome Atlas (TCGA) database, Human protein Atlas (HPA), and Genotype Tissue-Expression (GTEX) database. Analysis of IL-15 genomic alterations and protein expression in human organic tissues was analyzed by the cBioPortal database and HPA. The correlations between IL-15 expression and survival outcomes, clinical features, immune-associated cell infiltration, and ferroptosis/cuproptosis were analyzed using the TCGA, ESTIMATE algorithm, and TIMER databases. Gene Set Enrichment Analysis (GSEA) was performed to evaluate the biological functions of IL-15 in pan-cancer.

**Results:** The differential analysis suggested that the level of IL-15 mRNA expression was significantly downregulated in 12 tumor types compared with normal tissues, which is similar to the protein expression in most cancer types. The high expression of IL-15 could predict the positive survival outcome of patients with LUAD (lung adenocarcinoma), COAD (colon adenocarcinoma), COADREAD (colon and rectum adenocarcinoma), ESCA (esophageal carcinoma), SKCM (skin cutaneous melanoma), UCS (uterine carcinosarcoma), and READ (rectum adenocarcinoma). Moreover, amplification was found to be the most frequent mutation type of IL-15 genomic. Furthermore, the expression of IL-15 was correlated to the infiltration levels of various immune-associated cells in pan-cancer assessed by the ESTIMATE algorithm and TIMER database. In addition, IL-15 is positively correlated with ferroptosis/cuproptosis-related genes (ACSL4 and LIPT1) in pan-cancer. Levels of IL-15 were reported to be elevated in humans for 10–120 min following an acute exercise. Therefore, we hypothesized that the better prognosis of pan-cancer patients with regular exercise may be achieved by regulating level of IL-15.

**Conclusion:** Our results demonstrated that IL-15 is a potential molecular biomarker for predicting patient prognosis, immunoreaction, and ferroptosis/cuproptosis in pan-cancer and partly explained the anti-cancer effects of exercise.

## Introduction

Over time, the global rise in cancer incidence and mortality has corresponded with an increase in a range of cancer-related lifestyle factors, such as obesity and inactivity (Bray et al., 2018); thus, optimizing lifestyles and exercise (Anand et al., 2008) have been emphasized as tools for cancer prevention and management (Bourke et al., 2016; Gunnell et al., 2017). Exercise oncology-the application of sports medicine to cancer is a rapidly growing subspecialty within the field of clinical oncology, and the quantity and quality of research in this area are increasing (Jones and Alfano, 2013). Although substantial evidence has supported a link between exercise and reduced progression and mortality in several types of cancer: Exercise-induced myokines play an important role in increasing cytotoxicity and the infiltration of immune cells into the tumour on prostate cancer. Myokines released from activated skeletal muscle impaired growth and migration of PC(Pancreatic cancer) cells and enhanced PC cell death. However, the molecular mechanism between exercise and disease progression has not been fully elucidated. (Kim et al., 2021; Schwappacher et al., 2021). Research suggests that exercise could lead to a variety of physiological alterations, thereby reducing the risk of developing chronic disease. For example, the factors such as muscle secretome and catecholamines in serum will be regulated after exercise. (Jones and Alfano, 2013; Rönn et al., 2014; Bohlen et al., 2018; Chen et al., 2022). Preclinical evidence suggested that exercise may modulate levels of systemic factors such as local growth factors (IGF1), hormones (insulin and leptin), and inflammatory cytokines (IL-6, IL-15), which are known factors that have a possible impact on the cancer process (Kim et al., 2021). It has been determined that skeletal muscle is a significant source of IL-15 (rEF.146). After a single exercise session, IL-15 mRNA expression in skeletal muscle (Bohlen et al., 2018; Kim et al., 2021; Morrisson et al., 2021) was much greater in healthy volunteers than it was before the exercise (Riechman et al., 2004; Tamura et al., 2011). It was also widely reported that circulating levels of IL-15 were significantly higher after many kinds of exercise, which may benefit human health (Riechman et al., 2004; Nielsen et al., 2007; Tamura et al., 2011; Jones and Alfano, 2013; Bazgir et al., 2015; Crane et al., 2015; Palareti et al., 2016).

Interleukin 15 (IL-15) is a cytokine of the interleukin 2 (IL-2) family that has multiple functions similar to IL-2 in regulating the adaptive and innate immune systems (Bohlen et al., 2018). Although IL-15 is released in small amounts, the alpha receptor (IL15Rα), a transmembrane protein with a high affinity for IL-15, facilitates IL-15 transport from the endoplasmic reticulum through the cytoplasm and presents the IL-15/IL-15Rα complex on the cell surface (Nadeau and Aguer, 2019). Upon cell activation, IL-15 is secreted primarily by dendritic cells (DCs), macrophages, and monocytes (Do Thi et al., 2019); however, it can also be secreted by many other cell types, including endothelial cells, mesenchymal cells, and renal epithelial cells, but not by T cells or natural killer (NK) cells (Nielsen et al., 2007; Vilsmaier et al., 2021). With improved immune cell activation and migration, IL-15 is involved in anti-tumor immunity (Morrisson et al., 2021; Schwappacher et al., 2021; Vilsmaier et al., 2021). Many studies suggested that IL-15 is involved in tumor suppression by enhancing anti-tumor immunity (Crane et al., 2015; Dethlefsen et al., 2017; Bohlen et al., 2018; Liu et al., 2021). In recent years, many studies have been conducted to investigate the oncogenesis and development of pan-cancer to reveal the similarities and differences in cancer (Xu et al., 2021; Zhang et al., 2022). Therefore, it is of interest to further explore the spectrum of oncogenes in pan-cancer, but to date, there is no association analysis between IL-15 and pan-cancer.

Ferroptosis and cuproptosis are two forms of cell death induced by unbalanced ion homeostasis, which ferroptosis is driven by iron-dependent lipid peroxidation, while cuproptosis terms to intracellular copper accumulation triggers the aggregation and destabilization of proteins (Stockwell, 2022; Tang et al., 2022). A better understanding of the molecular determinants of the two cell death modes will contribute to innovate anticancer therapies (Lei et al., 2022; Tsvetkov et al., 2022). For example, much research has revealed that drugs targeting ferroptosis could kill cancer cells both *in vitro* and *in vivo* (Lei et al., 2022). Studies have reported CD8^+^ T cells could activate ferroptosis in tumor cells *in vivo* and further induce cancer cells death(32778143). But currently, rare studies have discovered the association between IL-15 and ferroptosis/cuproptosis-related genes in pan-cancers, which is wealthy to explore.

We have systematically investigated the role of IL-15 in human pan-cancer. We comprehensively investigated the different expression levels of IL-15 in tumor and normal control tissues by using The Cancer Genome Atlas (TCGA), Genotype-Tissue Expression (GTEX), and tumor-related databases. The predictive value of IL-15 on prognosis was also evaluated. Meanwhile, potential relationships between IL-15 mRNA expression levels and clinical features, DNA gene mutations, immune cells infiltration, and pathway regulation were evaluated in pan-cancer. This study highlights the multifaceted role of IL-15 in pan-cancer. Combined with its correlation with exercise, we provide a theoretical basis for exercise to prevent cancer and IL-15 as a pan-cancer therapeutic target.

## Methods

### Data processing and IL-15 expression analysis

Transcriptomic data and clinical features of tumor tissues were analyzed using UCSC Xena (https://xena.ucsc.edu/)) software (Chen et al., 2021). The GTEx portal (https://www.gtexportal.org/) was used to obtain Human normal tissue expression matrices. Strawberry Perl scripting software was applied to get the IL-15 expression data in 33 TCGA tumors and GTEx normal tissues (http://strawberryperl.com/, version 5.30.0.1). In addition, IL-15 expression in various tumor types and cell types was also assessed from the Human Protein Atlas database (https://www.proteinatlas.org/). The expression levels of IL-15 in healthy male and female tissues were presented by the Gganatgraph R program package. Expression data were cleared using log2 (TPM) to exclude missing data and duplicate values. R version 4.0.2 software was used (https://www.Rproject.org) to conduct the analysis. The Encyclopedia of Cancer Cell Lines (https://portals.broadinstitute.org/ccle/) was utilized to extract IL-15 mRNA expression in cell lines.

### Immunohistochemical tissue

The relative IL-15 protein expression data in pan-cancer were obtained in the Human Protein Atlas (https://www.proteinatlas.org/) including both normal tissue and pathology tissue (Zhang et al., 2022). The overall expression can be observed directly through tissue samples.

### Correlation analysis between genes in pan-cancer

The Timer database (https://cistrome.Shinyapps.io/Timer/) was utilized to analyze the relationship between the expression level of IL-15 and ferroptosis/cuproptosis-related genes in pan-cancerous tissues (Li et al., 2017). Ferroptosis: *SLC7A11, GPX4, CISD1, NFS1*, *NRF2*, *P53*, *VDACs*, *ACSL4*, and *NCOA4*; Cuproptosis: *CDKN2A*, *FDX1*, *DLD*, *DLAT*, *LIAS*, *GLS*, *LIPT1*, *MTF1*, *PDHA1*, and *PDHB*.

### Genomic alterations IL-15 in pan-cancer

The genetic alterations of IL-15 in the TCGA pan-cancer dataset were analyzed using the Bioportal database (http://www.cbioportal.org/) (Cerami et al., 2012). Data on the genetic alterations and mutation sites of IL-15 was gotten from the “OnCoprint”, “Summary of Cancer Types” and “Mutations” modules.

### Association analysis of IL-15 expression with tumor immune microenvironment in cancers

The lollipop plots were produced by R (3.6.3 version, GSVA packages) to show the correlation between IL-15 expression and immune cells, including aDC [activated DC]; B cells; CD8^+^ T cells; Cytotoxic cells; DC; Eosinophils; iDC [immature DC]; Macrophages; Mast cells; Neutrophils; NK CD56bright cells; NK CD56dim cells; NK cells; pDC [Plasmacytoid DC]; T cells; T helper cells; Tcm [T central memory]; Tem [T effector memory]; Tfh [T follicular helper]; Tgd [T gamma delta]; Th1 cells; Th17 cells; Th2 cells; Treg.

The Timer database (https://cistrome.Shinyapps.io/Timer/) was utilized to analyze the relationship between the expression level of IL-15 and the abundance of various immune-related cells in pan-cancerous tissues (Li et al., 2017). The expression of IL-15 in various cancer types was assessed by the “diff Exp” module in the Timer database.

The biological functions of IL-15 were analyzed using the Gene Set Enrichment Analysis (GSEA) (Luo et al., 2022a; Luo et al., 2022b; Sun et al., 2022). The GSEA online database (https://www.gsea-msigdb.org/gsea/downloads.jsp) was used to perform Kyoto Encyclopedia of Genes and Genomes (KEGG) analysis and the biological functions of IL-15 in pan-cancer were conducted *via* GSEA analysis using the R-packages “cluster Profiler”.

### Statistical analysis

Differences in expression and correlation between the two groups were assessed by Wilcoxon rank-sum test and the Spearman rank-sum test. Kaplan-Meier survival curves with the log-rank test were applied to survival analysis. Besides, Hazard ratios (HR) were calculated using Cox proportional risk regression models. In addition, statistical analysis was calculated by GraphPad Prism 9.0 and R software (4.0.2). *p* < 0.05 was considered statistically significant.

## Results

### Expression of IL-15 in normal tissue and pan-cancer

**FIGURE 1:**
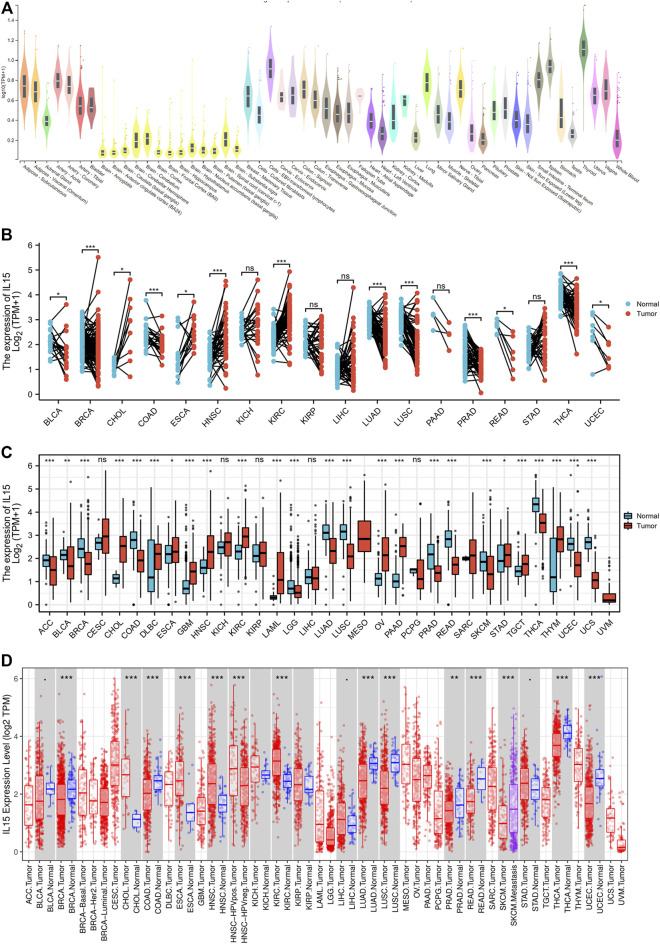
Differential expression pattern of IL-15. **(A)** IL-15 mRNA expression in normal tissues from GTEX data. **(B)** Differential IL-15 mRNA expression between paired samples in TCGA cancers. The red dot represents cancer samples, and the blue dot represents paired normal samples. **(C)** Differential IL-15 mRNA expression between TCGA cancers and GTEX normal tissues. The red column represents cancer samples, and the blue column represents normal samples. The normal group was normal tissue in TCGA and GTEX databases. **(D)** IL-15 mRNA expression in different cancer types in TIMER. The normal group was normal tissue in the TCGA database. *p < 0.05, **p < 0.01, and ***p < 0.001.

To assess the mRNA expression of IL-15 in human normal tissue, IL-15 expression was tested in physiological tissue of the GTEX dataset. IL-15 was overexpressed in the thyroid, adipose, endocrine tissues, bone marrow, and lung tissues (Figure 1A; Supplementary Figures S1A–C). As for IL-15 in normal cell types, endometrial stromal cells and monocytes express the highest level of IL-15 mRNA (Supplementary Figure S1D). The expression abundances of IL-15 in various tissues within male and female were also observed. Generally, no significant difference was found between gender and IL-15 mRNA expression levels (Supplementary Figure S1E). According to the Atlas database, IL-15 was decreased in almost all the human cell which in lines with analysis of the CCLE database except the Lymphoid cell line (Supplementary Figure S1F).

Then, *via* analyzing the RNA-seq data of TCGA and GTex databases, the expression of IL-15 in pan-cancer was assessed deeper. A significant expression difference of IL-15 was evaluated in 33 types of cancer in paired or unpaired samples except those without normal tissue data. In paired samples (Figure 1B), IL-15 expression was downregulated in BLCA (bladder urothelial carcinoma), BRCA (breast invasive carcinoma), COAD (colon adenocarcinoma), LUAD (lung adenocarcinoma), PRAD (prostate adenocarcinoma), READ (rectum adenocarcinoma), THCA (thyroid carcinoma), and UCEC (uterine corpus endometrial carcinoma), while upregulated in CHOL (cholangiocarcinoma), ESCA (esophageal carcinoma), HNSC (head and neck squamous cell carcinoma), and KIRC (kidney renal clear cell carcinoma). In unpaired samples (Figure 1C), IL-15 expression was considerably downregulated in ACC (adrenocortical carcinoma), BLCA, BRCA, COAD, LUAD, LUSC (lung squamous cell carcinoma), PRAD, READ, THCA, LGG (brain lower grade glioma), SKCM (skin cutaneous melanoma) and UCEC, while upregulated in CHOL, DLBC (lymphoid neoplasm diffuse large B-cell lymphoma), ESCA, GBM (glioblastoma multiforme), HNSC, KIRC, LAML (acute myeloid leukemia), OV (ovarian serous cystadenocarcinoma), PAAD (pancreatic adenocarcinoma), STAD (stomach adenocarcinoma), TGCT (testicular germ cell tumors), and THYM (thymoma) compared to control tissues. Next, the mRNA expression of IL-15 in human pan-cancer was further assessed in the TIMER database. IL-15 expression was increased in CHOL, ESCA, and HNSC, while IL-15 was significantly decreased in BRCA, ESCA, LUAD, LUSC, PRAD, READ, SKCM, THCA, UCEC, and COAD (Figure 1D) compared to the control group.

### Protein expression of the IL-15 in human tissues

**FIGURE 2:**
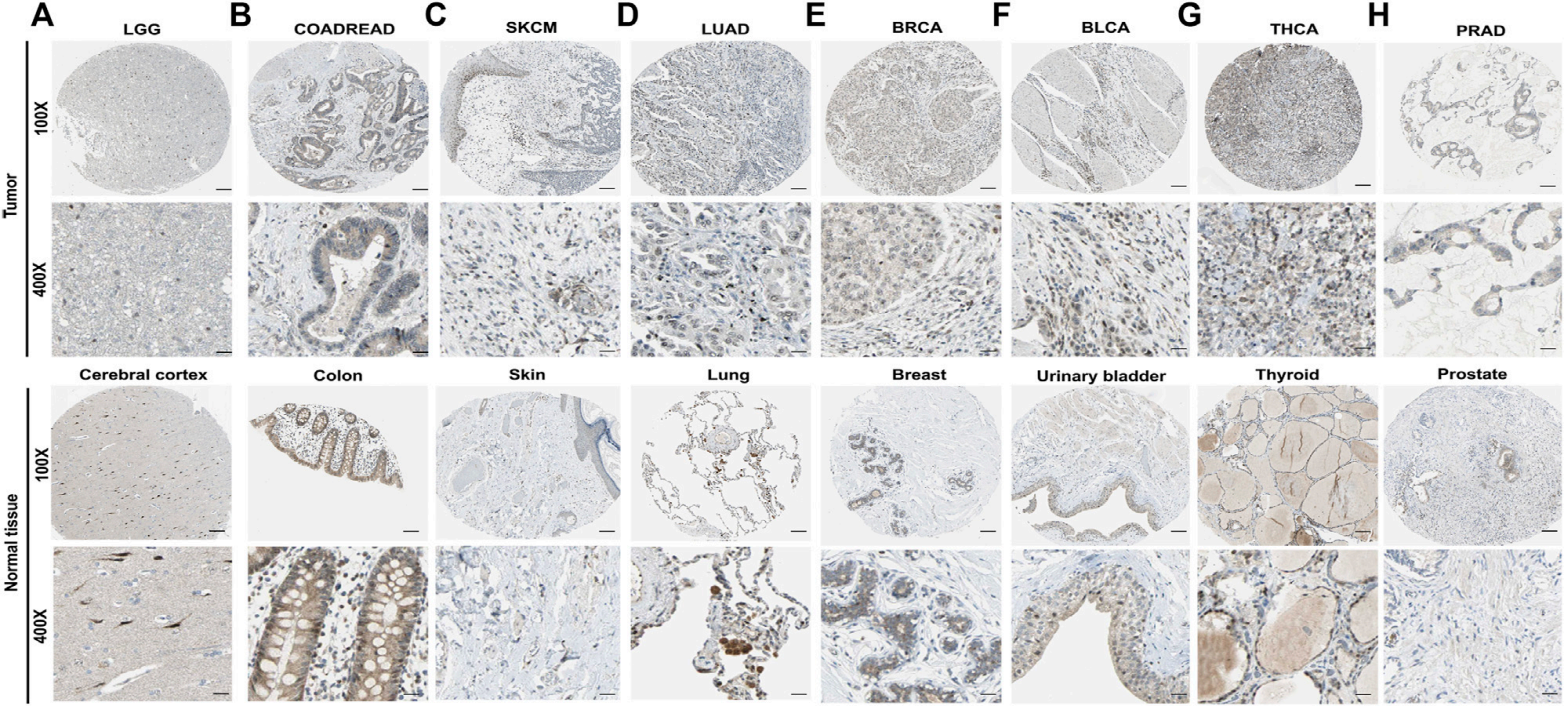
The protein expression level of IL-15 in human multiple cancer tissues **(A)** LGG, **(B)** COADREAD, **(C)** SKCM, **(D)** LUAD, **(E)** BRCA, **(F)** BLCA, **(G)** THCA, and **(H)** PRAD. Representative images of IL-15 expression in pan-cancer tissues are shown. Original magnification, ×100 and ×400. Scare bar = 200 μm or 50 μm.

The protein levels of IL-15 in pan-cancer were assessed by the Human Protein Atlas (HPA) (Figure 2). The protein levels of IL-15 were considerably downregulated in COADREAD (colon and rectum adenocarcinoma), LUAD, BRCA, and BLCA, while it was not obvious in LGG, SKCM, THCA, and PRAD compared with corresponding normal tissues.

### Prognostic assessment value of IL-15 in pan-cancer

**FIGURE 3:**
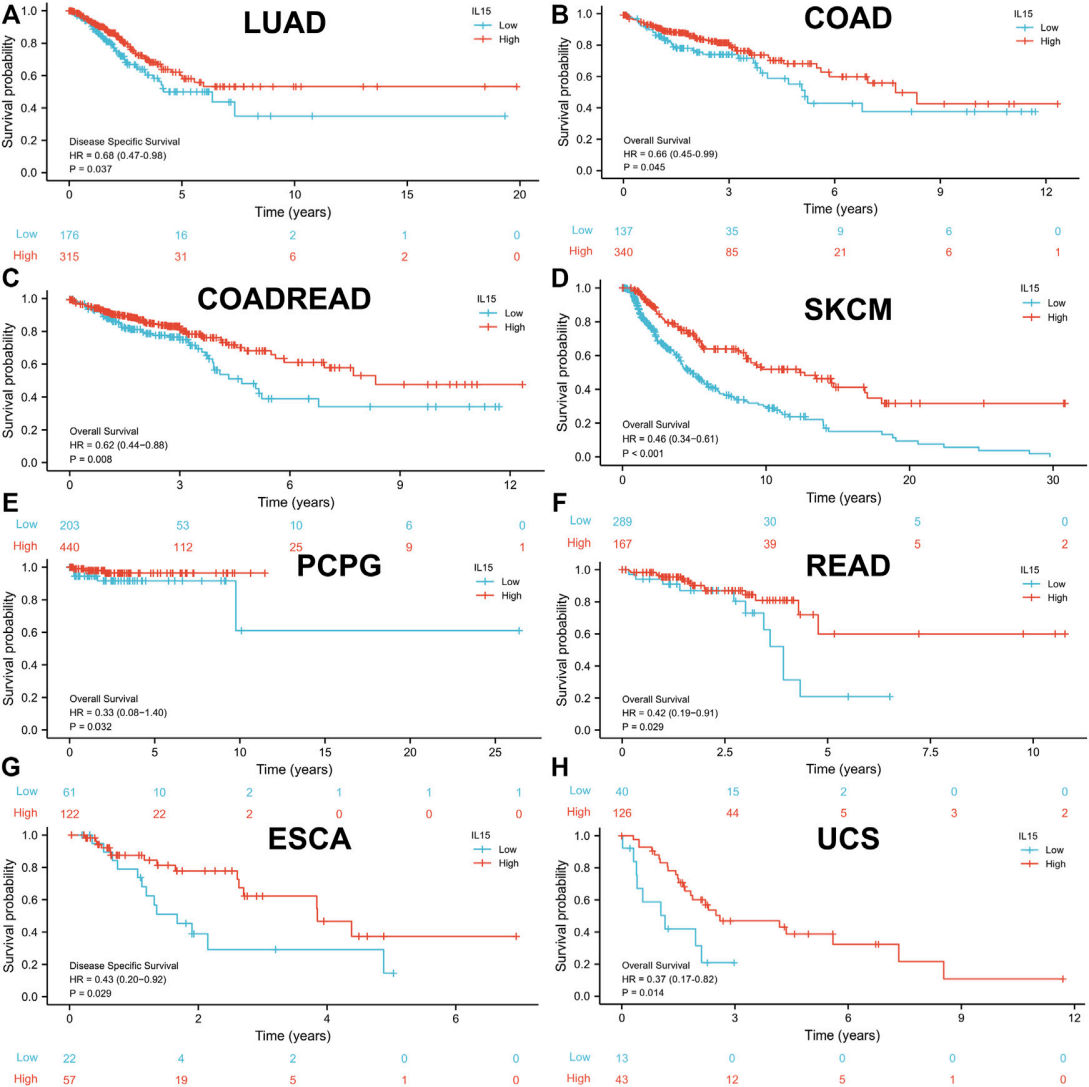
High expression of IL-15 promoted patient survival period. **(A and G)** Kaplan–Meier analysis of the association between IL-15 expression and DSS in LUAD and ESCA. **(B–F,H)** Kaplan–Meier analysis of the correlation between IL-15 expression and OS in COAD, COADREAD, SKCM, PCPG, READ, and UCS. The red line shows high IL-15 expression, and the blue line represents low IL-15 expression. OS, overall survival; DSS, disease-specific survival.

To investigate the prognostic assessment value of IL-15 in pan-cancer, Kaplan–Meier analysis and Cox proportional hazards model were performed to assess the relationship between IL-15 expression levels and patients’ survival period (Figure 3). The expression of IL-15 was positive correlated with OS (overall survival) in LUAD (*p* = 0.037), COAD (*p* = 0.045), COADREAD (*p* = 0.008), SKCM (*p* < 0.001), PCPG (*p* = 0.032), UCS (Uterine Carcinosarcoma, *p* = 0.014) and READ (*p* = 0.029) as a good prognostic marker performed by Cox proportional hazards regression model. On the contrary, IL-15 was regarded as a high-risk factor for OS of GBM, OSCC, LGG, THYM, LIHC, LAML, PAAD, and GBMLGG (Supplementary Table S1; Supplementary Figure S2). In addition, DSS (disease-specific survival) of ESCA (*p* = 0.029), COADREAD (*p* = 0.004), COAD (*p* = 0.047), LUAD (*p* = 0.037), READ (*p* = 0.017), and SKCM (*p* < 0.001) were also positive correlated with IL-15 expression level, while that of GBM (*p* = 0.005) and LGG (*p* < 0.001) was negative correlated with IL-15 levels. For PFI (Progression-Free Interval), the overexpressed mRNA level of IL-15 showed an adverse factor in LGG (*p* < 0.001), THYM (*p* = 0.019), GBM (*p* < 0.001), PRAD (*p* = 0.026) and OV (*p* = 0.022) (Supplementary Table S1). Kaplan–Meier curves for PFI indicated a positive correlation between IL-15 overexpression and good survival outcome in patients with LUAD (*p* = 0.048), COAD (*p* = 0.006), COADREAD (*p* < 0.001), SKCM (*p* < 0.001), UCS (*p* = 0.012) and READ (*p* = 0.002) (Supplementary Table S1). Therefore, we performed the following assessments on the good-survival patients positive correlated with high IL-15 expression, including LUAD, PCPG, COAD, READ, COADREAD, ESCA, SKCM, and UCS.

### Correlation analysis between IL-15 expression and clinicopathological phenotypes in pan-cancer

The correlation between the mRNA expression level of IL-15 and patients’ clinicopathological features progression was further investigated in pan-cancer. IL-15 expression was negatively correlated to tumor stage in LUAD, COAD, READ, COADREAD, ESCA and SKCM (Supplementary Figures S4A–G). Higher expression of IL-15 was found in the age≥65 years group in COAD (*p* < 0.05) (Supplementary Figure S3C). It was discovered that IL-15 expression level was not significantly associated with tumor treatment response, especially between CR and PR groups (Supplementary Figure S4). Then, it was found that the overexpression of IL-15 was significantly associated with tumor status in COAD, SKCM, and COADREAD (Supplementary Figures S4C,E,G). In the three cancer types, patients with lower IL-15 expression were with higher-level tumor status. These results suggest that IL-15 expression levels could impact the prognosis in LUAD, COAD, READ, COADREAD, ESCA, and SKCM patients.

### Genetic alteration analysis of IL-15 in pan-cancer

**FIGURE 4:**
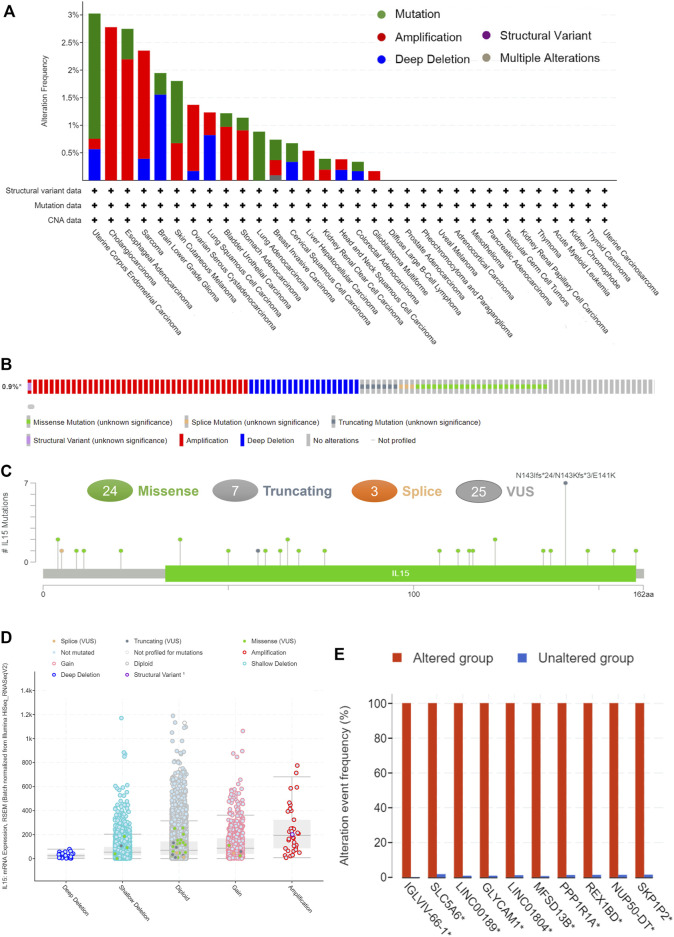
The genetic alterations of IL-15. **(A)** Alteration summary of IL-15 in TCGA pan-cancer datasets. **(B)** Summary of IL-15 structural variant, mutations, and copy-number alterations. **(C)** The mutation types, number, and sites of the IL-15 genetic alterations. **(D)** The alteration types of IL-15 in pan-cancer. **(E)**The related genes alteration frequency in the IL-15 altered group (Red) and unaltered group (blue).

Pan-cancer patients with uterine corpus endometrial carcinoma, sarcoma, cholangiocarcinoma, and esophageal adenocarcinoma owned the highest gene alteration rate of IL-15 (>2%) compared with the primary type in the cBioPortal database (Figure 4A). Three main types of frequent genetic alterations of IL-15 were missense mutation, amplification, and deep deletion (Figure 4B). Figure 4C further presented the types, sites, and case numbers of the IL-15 gene modification. IL-15 missense mutation was the main type of alteration, while splice alteration was detected in 3 cases. Amplification, gain function, and diploid were the Top-3 frequent putative copy-number alterations of IL-15 (Figure 4D). The gene alteration of IGLVIL-66-1, ALC5A6, Lnc00189, MFSD13B, PPP1R1A, REX1BD, NUP50-DT, and SKP1P2 was more frequent in the altered group than in the unaltered group (Figure 4E).

### Correlation of IL-15 expression with tumor immune microenvironment

**FIGURE 5:**
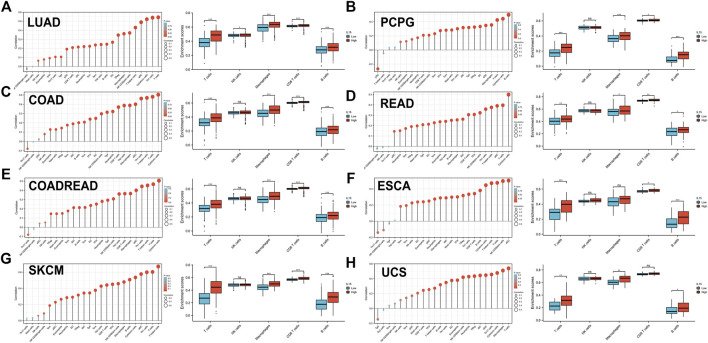
Correlation between IL-15 gene expression and tumor immune microenvironment in TCGA database. **(A–H)** Analysis of immune-associated cells infiltration with IL-15 expression in pan-cancer using lollipop diagrams and box plots. *p < 0.05, **p < 0.01, and ***p < 0.001.

To further assess the relationship between IL-15 and the human immune system, based on the ssGSEA algorithm and TIMER database, the relationship between IL-15 expression and the tumor immune microenvironment was evaluated (Figure 5). First of all, the relationship between IL-15 expression and immune-associated cells infiltration in pan-cancer was assessed using the ssGSEA algorithm. It was found that almost all the relative immune cells including aDC, B cells, CD8^+^ T cells, Cytotoxic cells, DC, Eosinophils, iDC, Macrophages, Neutrophils, NK CD56- cells, NK cells, T cells, T helper cells, Tcm, Tem, fh, Tgd, Th1 cells, and Treg, were positively correlated with IL-15 except Th17 cells, NK CD56^+^ cells, Th2 cells, Mast cells, and pDC presented by Lollipop diagrams. The boxplot displayed the statistical significance of T cells, NK cells, macrophages, CD8^+^ T cells, and B cells with low or high IL-15 expression., in which enrichment scores of the high IL-15 group were significantly higher than that of the low IL-15 group except for NK cells. Next, the TIMER database was used to further evaluate the relationship between immune-associated cells infiltration and IL-15 expression in pan-cancer. It was shown that IL-15 expression was significantly correlated with six types of infiltrating immune-associated cells including B cells, CD8^+^T cells, CD4^+^T cells, neutrophils, macrophages, and dendritic cells in LUAD, COAD, SKCM, ESCA, and USC, except PCPG (Supplementary Figure S4).

### Biological function of IL-15 in cancer

**FIGURE 6:**
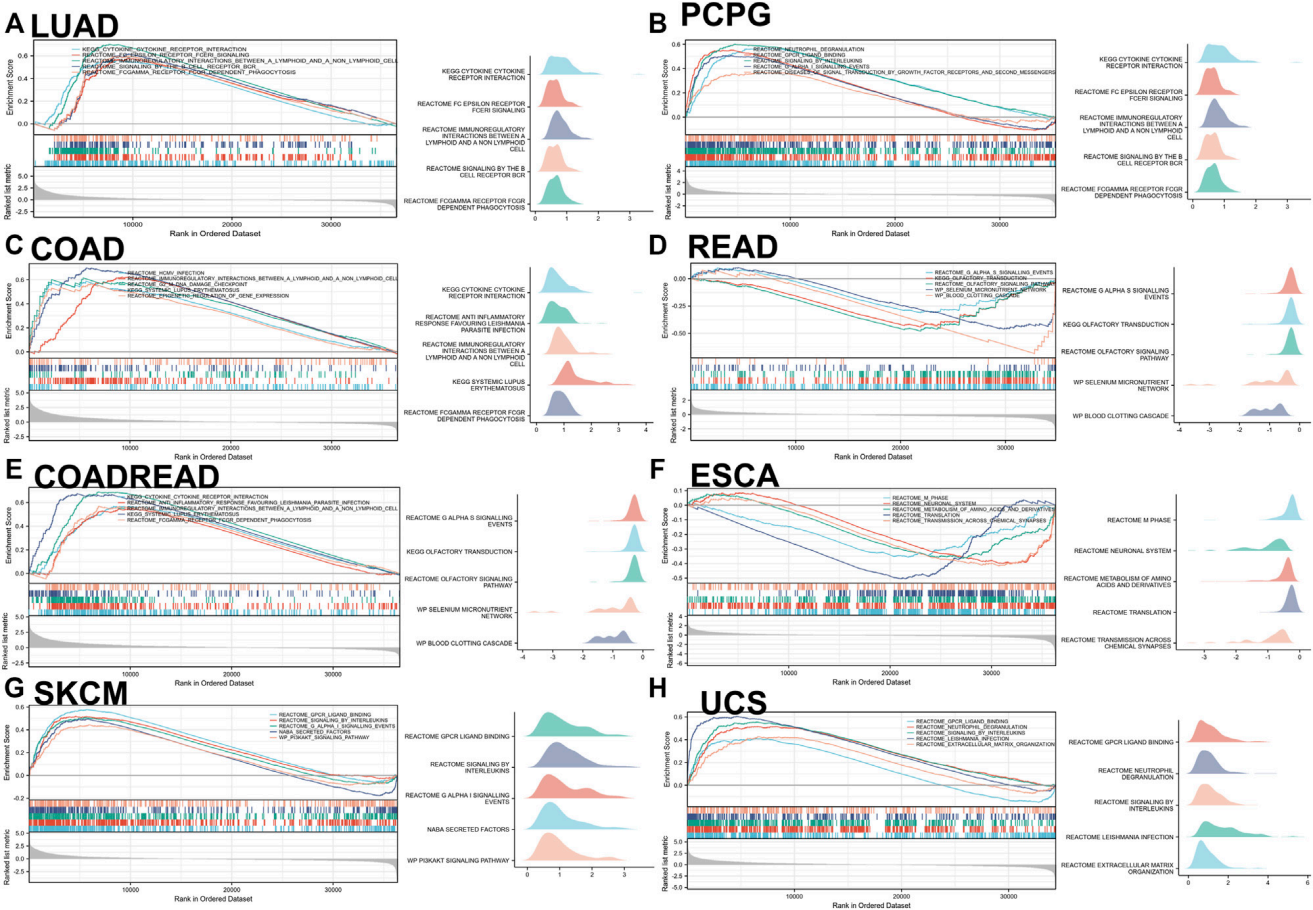
GSEA analysis in KEGG signature of IL-15 in LUAD **(A)**, PCPG **(B)**, COAD **(C)**, READ **(D)**, COADREAD **(E)**, ESCA **(F)**, SKCM **(G)**, and UCS **(H)**. The left panel: Different color curves show different functions or pathways (Top 5). The peak of the upward and downward curve represents the positive and negative regulation of IL-15, respectively. Score, enrichment score. The right panel: Summary of GSEA plots of representative data is presented. The horizontal axis is the degree of correlation, and the vertical axis is the corresponding pathway.

The main (Top-5) biological processes affected by IL-15 were explored by GSEA analysis in pan-cancer. Based on KEGG gene sets analysis, the data suggested that IL-15 positively regulated signaling pathways in LUAD, PCPG, COAD, READ, COADREAD, ESCA, SKACM, and UCS (Figure 6). Cytokine receptor interaction and Gαs signaling were the most common signaling pathways of IL-15 for pan-cancer, followed by olfactory transduction, neutrophil degranulation, immunoregulatory interactions pathway. In addition, GPCR(G Protein-Coupled Receptor) ligand binding, infection, phagocytosis, and DNA damage checkpoint pathways were all involved in IL-15 biology function in pan-cancer biological analysis.

### Correlation of IL-15 expression with ferroptosis-related genes and survival analysis

**FIGURE 7:**
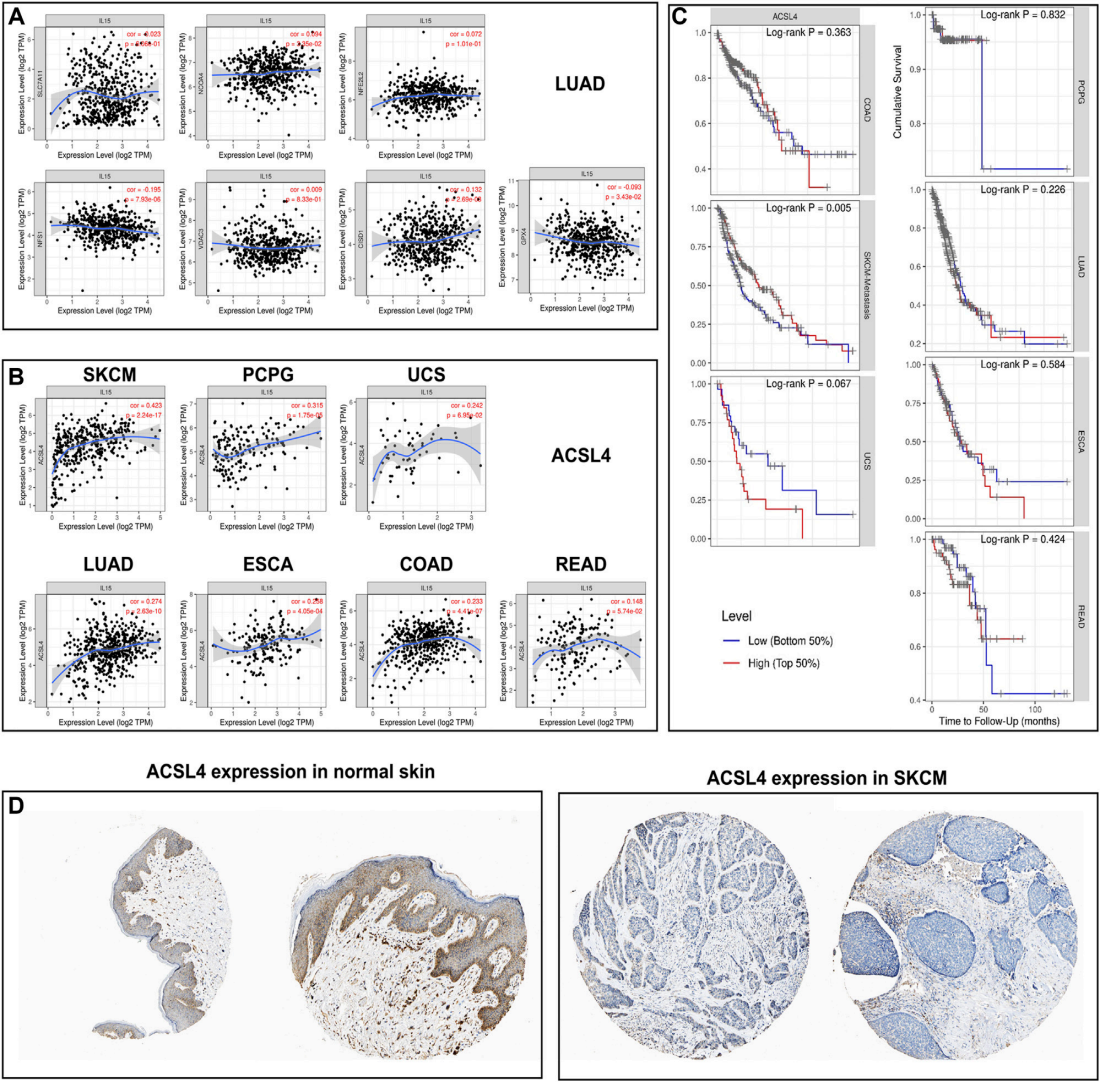
Correlation and survival analysis for IL-15 and ferroptosis-related genes in pan-cancers **(A)** Correlation between IL-15 and ferroptosis-related genes in LUAD. **(B)** Correlation between IL-15 and *ACSL4* in pan-cancers. **(C)** Survival analysis for *ACSL4* in pan-cancers. **(D)** Protein expression of *ACSL4* in SKCM and normal skin tissue.

We first analyzed the expression of ferroptosis-related genes with IL-15 in LUAD, the largest sample size, and our results only found a correlation between *ACSL4* and IL-15 (Figure 7). We further analyzed the expression of *ACSL4* in pan-cancer, and we found a strong and significant positive correlation between ACSL4 and IL-15 in SKCM patients, and a general level of positive correlation in *PCPG, UCS, ESCA, COAD,* and *LUAD* (*p* less than 0.05), while the correlation was not strong in READ. Further, we analyzed the relationship between *ACSL4* and overall survival, and we found that in SKCM, high expression of *ACSL4* significantly increased patient survival (*p* less than 0.05). The above results suggest that the improvement of overall survival in SKCM by IL-15 may be achieved by promoting the expression of iron death-related genes. In addition, low expression of *ACSL4* protein was found in SKCM and *ACSL4* protein was relatively higher in normal skin tissue.

### Correlation of IL-15 expression with cuproptosis-related genes and survival analysis

**FIGURE 8:**
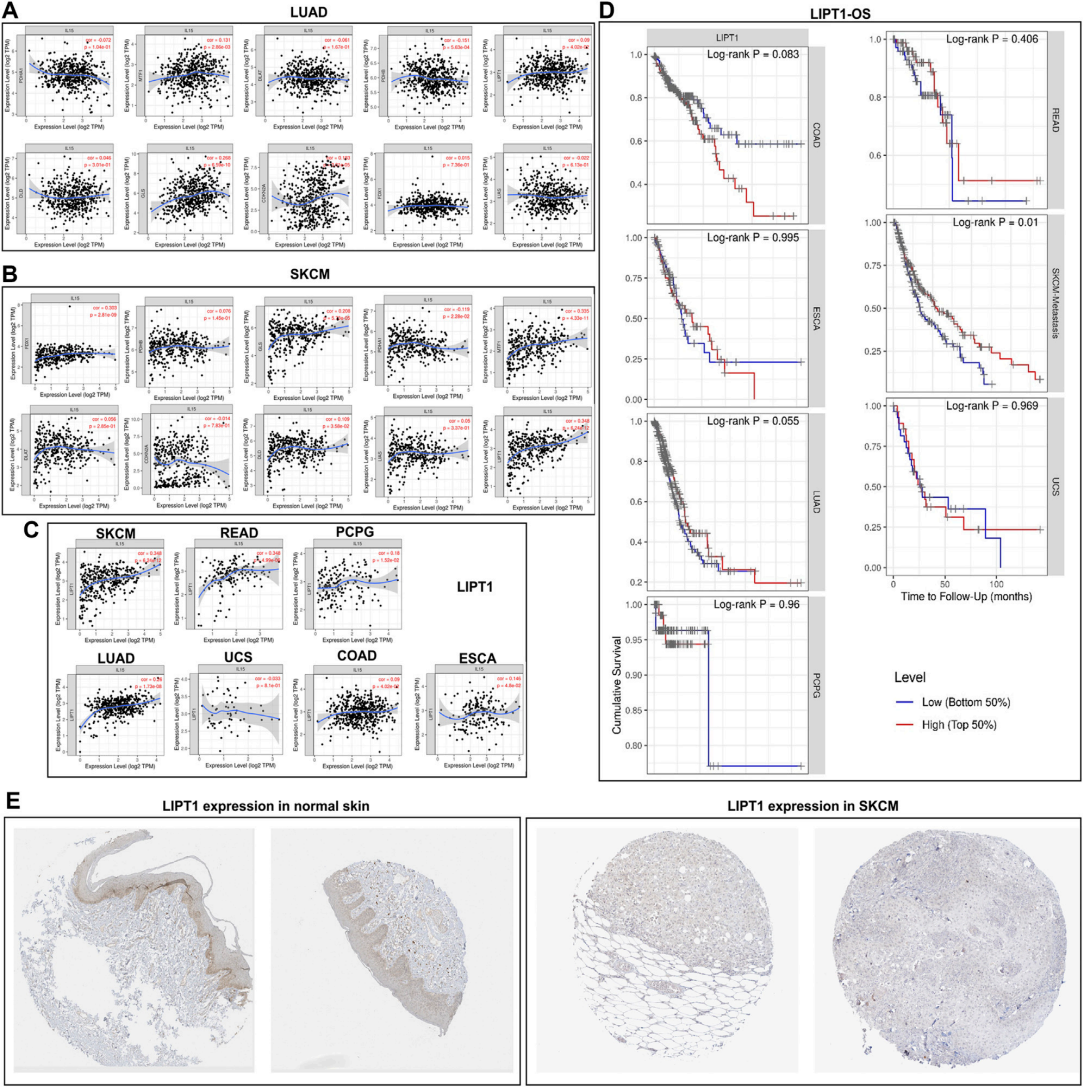
Correlation and survival analysis for IL-15 and cuproptosis-related genes in pan-cancers **(A)** Correlation between IL-15 and cuproptosis-related genes in LUAD. **(B)** Correlation between IL-15 and cuproptosis -related genes in SKCM. **(C)** Correlation between IL-15 and *LIPT1* in pan-cancers. **(D)** Survival analysis for *LIPT1* in pan-cancers. **(E)** Protein expression of *LIPT1* in SKCM and normal skin tissue.

We first analyzed the expression of cuproptosis-related genes with IL-15 in LUAD and SKCM, which had the largest sample size, and our results found a correlation between *LIPT1*, *FDX1*, *MTF1*, and IL-15, and *LIPT1* was the strongest (R = 0.348) (Figure 8). Further, we analyzed the expression of LIPT14 in pan-cancer, and we found that LIPT1 and IL-15 had a strong and significant positive correlation in SKCM and READ, and a general level of positive correlation in LUAD, ESCA, PCPG (*p* less than 0.05), but not with UCS, COAD. We further analyzed the relationship between *ACSL4* and overall survival, and we found that in SKCM, high expression of *LIPT1* significantly increased the survival rate of patients (*p* less than 0.05), meanwhile, in LUAD, high expression of *LIPT1* also show general prognostic impact (*p* = 0.055). The above results suggest that IL-15 improves the overall survival of SKCM, and LUAD may be reached by promoting the expression of cuproptosis-related genes. Furthermore, low expression of *LIPT1* protein was found in SKCM and *LIPT1* protein was relatively higher in normal skin tissue.

## Discussion

Numerous studies have shown that circulating levels of IL-15 are elevated in humans for 10–120 min following an acute exercise (Riechman et al., 2004; Tamura et al., 2011; Crane et al., 2015). For example, Regular endurance training for 12 weeks led to a 40% rise in the amount of IL-15 protein in the basal skeletal muscle (Rinnov et al., 2014). Weightlifting increased circulating IL-15 levels in untrained and trained young subjects during and immediately after exercise (Nielsen et al., 2007; Bazgir et al., 2015; Nadeau and Aguer, 2019; Kim et al., 2021). Recently, IL-15 has emerged as a promising cytokine for the treatment of cancer (Berger et al., 2019; Ligibel et al., 2019; Xiao et al., 2019; Schwappacher et al., 2021; Pereira et al., 2022). IL-15 is also a key factor in the development, proliferation, and activation of NK cells and CD8^+^ T cells (Berger et al., 2019; Do Thi et al., 2019; Fiore et al., 2020; Liu et al., 2021), which are able to destroy cancer cells in the tumor microenvironment. Several current products include ALT-803 ALT-803 (Hu et al., 2018), P22339 (Hu et al., 2018), chimeric IL-15 apolipoprotein A-I (Ochoa et al., 2018), or NKTR-255 (Miyazaki et al., 2021), illustrating the promise of IL-15 in cancer therapy. Recently, some completed clinical trials have reported usage of recombinant human single-chain IL-15 and IL-15 superagonist in cancer treatment (Zhang et al., 2021). Conlon et al. utilized recombinant human single-chain IL-15 to treat metastatic malignant melanoma or renal cell cancer patient with maximum tolerated dose 0.3 ug/kg (Conlon et al., 2015). The results indicated IL-15 could dramatically mediate the NK cells in blood. In the following study by Conlon et al.and Miller et al., the results showed a better mediated effect to NK cells and CD8^+^ cells when the maximum tolerated dose up to 2 ug/kg (Miller et al., 2018; Conlon et al., 2019). However, no studies have explored IL-15 in pan-cancer, limiting its possible clinical application, and the present study has explored the relevance by focusing on both prognostic and immunological directions.

It was found in this study that IL-15 is highly expressed in the thyroid, intestine, bone marrow, and lymphatic system, while it is less expressed in tissues such as skin and pancreas and varies independently of sex. It was also decreased in most tumor cell lines and was highly expressed in epithelial cells and monocytes, which are similar to the previous reports (Van Acker et al., 2018; Nadeau and Aguer, 2019; Xiao et al., 2019; Liu et al., 2021). IL-15 gene expression was found to be downregulated in BLCA, BRCA, COAD, LUAD, PRAD, READ, THCA, and UCEC in both matched and unmatched specimens in the TCGA database, GTex database, and TIMER database. We further examined IL-15 expression in tissues and found that the relative expression of IL-15 protein was downregulated in COADREAD, LUAD, BRCA, and BLCA. Currently, rare studies have checked the IL-15 expression in pan-cancer using tissue microassays except in those databases. Cierna et al. (2021) found that decreased levels of circulating IL-15 suggested PD-L1 overexpression in tumors of primary breast cancer patients and poor prognosis. Krizia et al. (Cierna et al., 2021) reported that higher IL-15 levels would lead to better outcomes in prostate cancer, which is consistent with our findings. Margolin et al. (2018) recently found that the injection of IL-15N72D: IL-15RαSu/IgG1 Fc complex could reduce cancer growth, induce NK cell expansion and improve survival in patients with incurable advanced melanoma, renal cell, non-small cell lung, and head and neck cancer. The above results suggest that IL-15 plays an important role in the development of human cancers.

Next, we sought to analyze the relationship between mRNA expression levels of IL-15 and the prognosis of patients with cancer. Kaplan-Meier survival analysis showed that OS in LUAD, COAD, COADREAD, SKCM, UCS, and READ, OS in LUAD, COAD, COADREAD, SKCM, UCS and READ, PFI in ESCA, COADREAD, COAD, LUAD, READ, and DSS in SKCM were positively correlated with IL-15 expression, with statistically significant differences. In addition, we found that IL-15 was significantly lower in COAD, SKCM, and COADREAD with worse clinical staging compared to patients with lower staging. These results may confirm that IL-15 plays a protective role in human cancers. However, we also found that mRNA expression levels of IL-15 played a cancer-promoting role in GBM, OSCC, LGG, THYM, LIHC, LAML, PAAD, and GBMLGG. These suggest that the mechanism of IL-15 in cancers are complex and IL-15 may play a negative role in certain cancer types in a specific condition, which will need to be further tested in the future (Waldmann, 2014; Fiore et al., 2020; Cierna et al., 2021). For example, overexpression of IL-15 mRNA is associated with clinical staging and metastasis in cutaneous T-cell lymphoma (Döbbeling et al., 1998), but we found from the Kaplan-Meier database that IL-15 is more likely to be a protective factor for patients with SKCM. In addition, clinical studies have reported a correlation between high intra-tumor IL-15 concentrations and poor clinical outcomes in patients with lung cancer (Seike et al., 2007), which is contrary to our results. We suspect that the discrepancy between our online database results and the reported data may be due to the different methods used to detect IL-15 expression. Gene expression of IL-15 in lung adenocarcinoma, whereas mRNA and protein expression of IL-15 was detected in the Kaplan-Meier and Atlas databases. Therefore, the result of this study should be further validated in corresponding cancer samples for deeper investigation.

The IL-15 gene is located on chromosome 4q31 and encoded the 14–15 kDa glycoprotein-IL-15(54). IL-15 deficiency, due to mutations in its gene, has been extensively studied in many diseases, such as liver injury (Hou et al., 2012), diabetes (Liu et al., 2017) and allergy (Mathias et al., 2017). However, there are rare studies on the alteration of the IL-15 gene in human tumors. Therefore, we used the cBio-portal database to reveal the fact that amplification is the greatest frequency of IL-15 changes in pan-cancer. Alterations in IGLVIL-66-1, ALC5A6, Lnc00189, MFSD13B, PPP1R1A, REX1BD, NUP50-DT, and SKP1P2 were found to co-exist among IL-15 mutations. However, by our assay, we found that IL-15 variants do not seem to affect the prognosis of cancer patients (data not presented). Furthermore, studies have reported that if IL-15 deficiency leads to a range of problems (Gillgrass et al., 2014; Mathias et al., 2017; Van Acker et al., 2018; Schwappacher et al., 2021), suggesting that these variants may not affect the functional profile of IL-15 in cancer.

Due to a marked reduction in the number of peripheral lymphocytes, NK cells, T cells in IL-15-deficient mice (Grabstein et al., 1994; Miller et al., 2018), we believed that IL-15 may play an important role in the immune regulation of human tumors. Firstly, we used the ssGSEA algorithm in the TCGA database and found that IL-15 was positively associated with a range of immune cells including aDC, B cells, CD8^+^ T cells, Cytotoxic cells, DC, Eosinophils, iDC, Macrophages, Neutrophils, NK CD56^-^ cells, NK cells, T cells, T helper cells, Tcm, Tem, fh, Tgd, Th1 cells, and Treg. Only NK CD56^+^ cells, Th2 cells, Mast cells, and pDC were not significantly positively correlated with IL-15 expression. Timer database mining further revealed that IL-15 expression was significantly correlated with infiltration levels of a variety of immune-related cells, including B cells, CD8^+^T cells, CD4^+^T cells, neutrophils, macrophages, and dendritic cells in LUAD, COAD, SKCM, ESCA, and USC. The role of IL-15 in the human immune system has been studied in recent years. In mouse models, exogenous IL-1t5 treatment can reverse the downregulation of NK cell and T cell activity in IL-15 deficient mice (Kennedy et al., 2000). In terms of the cancer microenvironment, systemic IL-15 treatment reduced tumor growth, metastasis, and recurrence by increasing the cytotoxic effects of NK cells in mice inoculated with a lung and breast cancer models (Tang et al., 2008; Gillgrass et al., 2014). A preclinical study showed reduced tumor growth and increased immune cell infiltration in a mouse model of prostate cancer with IL-15 overexpression 2.5-fold and 2.7-fold higher numbers of CD8^+^ T cells and NK cells, respectively than in mice in the model without IL-15 injection (Morris et al., 2014). Our study suggested that IL-15 could also be useful in LUAD, COAD, SKCM, ESCA, and USC by improving the infiltration of the immune cells in tumors.

Furthermore, it has been suggested that IL-15 plays an important role in modulating lipid metabolism and glucose metabolism (Nadeau and Aguer, 2019; Kim et al., 2021). In mice, high levels of circulating IL-15 prevented abnormal glucose tolerance and insulin resistance induced by the high-fat diet. Overexpression of muscle-specific IL-15 in mice and supraphysiological IL-15 treatment in rats resulted in lower respiratory exchange rates and higher whole-body fatty acid oxidation, showing a greater tendency to use lipids as a high-energy fuel (Almendro et al., 2006; Morris et al., 2014). The series of abnormal expressions of lipid metabolism and gluconeogenic signaling pathways that may be induced by the reduction of IL-15 in pan-cancer may lead to the metabolic dysfunction of the body in the development of cancer (Crane et al., 2015; Bohlen et al., 2018; Nadeau and Aguer, 2019). This can at least partly explain why the upregulated IL-15 gene expression can be a risk factor in OS of GBM, OSCC, LGG, THYM, LIHC, LAML, PAAD, and GBMLGG in our results. Moreover, GPCR ligand binding, infection, phagocytosis, and DNA damage checkpoints were found to be IL-15 mediated pathways in pan-cancer, which deserved further investigation.

Finally, we analyzed the relationship between IL-15 and ion-induced death and their impact on clinical prognosis. Our results show that IL-15 is positively associated with ferroptosis/cuproptosis-related genes in various types of tumors, such as *LIPT1, ACSL4*. High expression of these genes can improve the prognosis of patients with SKCM and LUAD, which suggests that IL-15 may be able to kill cancer cells by activating the process of ferroptosis/cuproptosis. The literature reports that loss *ACSL4* will promote cancer progress and enhanced *ACSL4* expression will result in better clinical outcomes in tumors (Liao et al., 2022). Furthermore, high LIPT1 expression was reported to be a good prognostic factor in SKCM (Lv et al., 2022), which is in line with our work. IL-15 may be in synergy with ferroptosis/cuproptosis inducers for tumor treatment in the future.

Further studies can continue to delve into the evidence of potential associations between IL-15 expression and DNA mismatch repair system, microsatellite instability, ferroptosis/cuproptosis, or tumor mutation burden, and explore the possibility that IL-15 may influence the response of cancer patients to immune checkpoint therapy, which will contribute to further understanding of the mechanisms of immunotherapy for cancer treatment. In addition, pharmacological target exploration can be performed to search for drugs that can target or synergize IL-15 and contribute to clinical cancer treatment.

## Conclusion

This study systematically evaluated the characteristics of IL-15 in various aspects, including expression pattern, survival prognosis, genetic mutation, tumor immune microenvironment, ferroptosis/cuproptosis, and signaling pathway. Exercise-induced IL-15 might serve as a potential candidate for multiple-cancer treatments since it showed low expression in multiple cancers and predicted a better prognosis in cancer patients. Moreover, the aberrant IL-15 expression may be related to the tumour immune microenvironment in multiple types of cancer. This study highlights the positive roles of IL-15 in pan-cancer and provides data-based insights for the application of IL-15 in cancer treatment.

## Data Availability

The original contributions presented in the study are included in the article/Supplementary Material, further inquiries can be directed to the corresponding authors.

## Glossary

ACC: Adrenocortical carcinoma
BLCA: Bladder urothelial carcinoma
BRCA: Breast invasive carcinoma
CHOL: Cholangiocarcinoma
COAD: Colon adenocarcinoma
COADREAD: Colon and rectum adenocarcinoma
DCs: Dendritic cells
DLBC: Lymphoid neoplasm diffuse large B-cell lymphoma
DSS: Disease-specific survival
ESCA: Esophageal carcinoma
GBM: Glioblastoma multiforme
GSEA: Gene Set Enrichment Analysis
GTEX: Genotype Tissue-Expression
HNSC: Head and neck squamous cell carcinoma
HPA: Human protein Atlas
HR: Hazard ratios
IL-15: Interleukin 15
KEGG: Kyoto Encyclopedia of Genes and Genomes
KIRC: Kidney renal clear cell carcinoma
LAML: Acute myeloid leukemia
LGF: Local growth factors
LGG: Brain lower grade glioma
LUAD: Lung adenocarcinoma
LUSC: Lung squamous cell carcinoma
NK: Natural killer
OS: Overall survival
OV: Ovarian serous cystadenocarcinoma
PAAD: Pancreatic adenocarcinoma
PFI: Progression-Free Interval
PRAD: Prostate adenocarcinoma
READ: Rectum adenocarcinoma
SKCM: Skin cutaneous melanoma
STAD: Stomach adenocarcinoma
TCGA: The Cancer Genome Atlas
TGCT: Testicular germ cell tumors
THCA: Thyroid carcinoma
THYM: Thymoma
UCEC: Uterine corpus endometrial carcinoma
UCS: Uterine carcinosarcoma
