# Supplementary material for: Exercise-induced IL-15 acted as a positive prognostic implication and tumor-suppressed role in pan-cancer
Source: Front Pharmacol. 2022 Nov 17;13:1053137. doi: 10.3389/fphar.2022.1053137 (PMC9712805; doi:10.3389/fphar.2022.1053137)
Supplement: Supplementary file 1 [file Table1.DOCX]

**SUPPLEMENTARY TABLE 1**. Cox proportional hazards model of IL-15 expression in pan-cancer.

| Cancer | OS | | PFI | | DSS | |
| --- | --- | --- | --- | --- | --- | --- |
|  | *P* | HR | *P* | HR | *P* | HR |
| COADR  ACC  BLCA  BRCA  CESC  CHOL  COAD  DLBC  ESCA  GBM  HNSC  KICH  KIRC  KIRP  LAML  LGG  LIHC  LUAD  LUSC  MESO  OV  PAAD  PCPG  PRAD  READ  SARC  SKCM  STAD  TGCT  THCA  THYM  UCEC  UCS  UVM | **0.008**0.2660.386  0.0910.301  0.278**0.045**  0.0930.236  **0.008**0.346  0.3470.373  0.321**0.001**  **0.0000.049**  0.0580.074  0.7560.241  **0.0380.032**  0.205**0.029**  0.194**0.000**  0.098  0.999  0.074**0.003**  0.253**0.014**  0.105 | **0.62(0.44-0.88)**  1.58(0.71-3.51)  0.88(0.65-1.18)  0.76(0.55-1.05)  0.77(0.47-1.26)  0.54(0.18-1.65)  **0.66(0.45-0.99)**  3.61(0.81-16.15)  1.36(0.82-2.25)  **1.73(1.15-2.60)**  0.87(0.65-1.16)  1.95(0.49-7.79)  0.86(0.61-1.20)  0.73(0.40-1.35)  **2.04(1.33-3.14)**  **2.27(1.62-3.19)**  **1.42(1.00-2.02)**  0.76(0.57-1.01)  1.36(0.97-1.90)  1.08(0.67-1.72)  1.17(0.90-1.52)  **1.75(1.03-2.97)**  **0.33(0.08-0.40)**  2.28(0.64-8.18)  **0.42(0.19-0.91)**  0.76(0.50-1.15)  **0.46(0.34-0.61)**  1.34(0.95-1.89)  /  0.38(0.13-1.10)  **23.94(2.88-198.81)**  0.79(0.52-1.18)  **0.37(0.17-0.82)**  2.00(0.87-4.63) | **0.000**0.358  0.1770.093  0.1890.331  **0.006**0.105  0.287**0.000**  0.3110.438  0.4260.310  /  **0.00**  0.058**0.048**  0.0960.337  **0.022**0.226  0.478**0.026**  **0.002**0.121  **0.000**0.669  0.0610.151  **0.019**0.332  **0.012**0.102 | **0.57(0.42-0.77)**  1.48(0.64-3.44)  0.81(0.60-1.10)  0.69(0.45-1.06)  0.72(0.44-1.18)  1.56(0.64-3.84)  **0.61(0.43-0.87)**  2.97(0.80-11.08)  1.29(0.81-2.06)  **2.46(1.57-3.83)**  0.85(0.62-1.17)  0.62(0.18-2.10)  0.88(0.64-1.20)  1.32(0.77-2.26)  /  **1.70(1.28-2.25)**  1.33(0.99-1.79)  **1.34(1.00-1.78)**  1.27(0.96-1.67)  0.75(0.42-1.35)  **1.37(1.05-1.79)**  1.27(0.86-1.89)  0.74(0.32-1.71)  **1.76(1.07-2.88)**  **0.31(0.15-0.65)**  1.30(0.93-1.81)  **0.56(0.45-0.71)**  0.938(0.699−1.258)  0.55(0.30-1.03)  0.65(0.36-1.17)  **3.10(1.21-7.95)**  1.21(0.83-1.76)  **0.42(0.21-0.82)**  1.87(0.88-3.98) | **0.004**0.425  0.2890.054  0.1580.389  **0.047**0.065  0.170**0.005**  0.3460.287  0.4060.305  /  **0.000**  0.051**0.037**  0.1940.473  0.1300.130  0.4780.311  **0.017**0.286  **0.000**0.154  0.999  /  0.9990.131  0.0520.105 | **0.52(0.33-0.81)**  0.76(0.40-1.48)  0.83(0.58-1.18)  0.73(0.53-1.01)  0.66(0.37-1.18)  0.61(0.19-1.89)  **0.60(0.36-0.99)**  8.44(0.88-81.25)  0.66(0.36-1.20)  **1.69(1.17-2.44)**  1.18(0.84-1.67)  2.44(0.47-12.58)  0.85(0.58-1.25)  0.67(0.31-1.44)  /  **2.28(1.59-3.26)**  1.56(1.00-2.42)  **0.68(0.47-0.98)**  1.33(0.86-2.05)  1.25(0.68-2.29)  1.25(0.94-1.65)  1.55(0.88-2.75)  0.55(0.11-2.84)  3.11(0.35-27.92)  **0.21(0.06-0.75)**  0.78(0.49-1.24)  **0.43(0.31-0.59)**  1.30(0.91-1.85)  /  /  /  1.62(0.87-3.04)  0.47(0.22-1.01)  2.06(0.86-4.92) |

OS, overall survival; PFI, progression-free interval; DSS, disease-specific survival. *P*, *P*-value; HR, hazard ratio.
