# Supplementary material for: Exercise-induced IL-15 acted as a positive prognostic implication and tumor-suppressed role in pan-cancer
Source: Front Pharmacol. 2022 Nov 17;13:1053137. doi: 10.3389/fphar.2022.1053137 (PMC9712805; doi:10.3389/fphar.2022.1053137)
Supplement: Supplementary file 2 [file DataSheet2.DOCX]

Scripts for Veen graph

# veen

library(tidyverse)

library(ggplot2)

## 2 group ##

# a <- dat[,1][!is.na(dat[,1])]

# b <- dat[,2][!is.na(dat[,2])]

# A <- setdiff(a, b)

# B <- setdiff(b, a)

# AB <- intersect(a, b)

# items <- list(A = A, B = B, AB = AB)

## 3 group ##

# a <- dat[,1][!is.na(dat[,1])]

# b <- dat[,2][!is.na(dat[,2])]

# c <- dat[,3][!is.na(dat[,3])]

# A <- setdiff(a, union(b, c))

# B <- setdiff(b, union(a, c))

# C <- setdiff(c, union(a, b))

# AB <- setdiff(intersect(a, b), c)

# AC <- setdiff(intersect(a, c), b)

# BC <- setdiff(intersect(b, c), a)

# ABC <- intersect(intersect(a, b), c)

# items <- list(A = A, B = B, C = C,

# AB = AB, AC = AC, BC = BC,

# ABC = ABC)

## 4 group ##

# A <- setdiff(a, union(union(b, c), d))

# B <- setdiff(b, union(union(a, c), d))

# C <- setdiff(c, union(union(b, a), d))

# D <- setdiff(d, union(union(b, a), c))

# AB <- setdiff(intersect(a, b), union(c, d))

# AC <- setdiff(intersect(a, c), union(b, d))

# AD <- setdiff(intersect(a, d), union(c, b))

# BC <- setdiff(intersect(c, b), union(a, d))

# BD <- setdiff(intersect(d, b), union(c, a))

# CD <- setdiff(intersect(c, d), union(a, b))

# ABC <- setdiff(intersect(intersect(a, b), c), d)

# ABD <- setdiff(intersect(intersect(a, b), d), c)

# ACD <- setdiff(intersect(intersect(a, d), c), b)

# BCD <- setdiff(intersect(intersect(d, b), c), a)

# ABCD <- intersect(intersect(intersect(a, b), c), d)

# items <- list(A = A, B = B, C = C, D = D, AB = AB, AC = AC,

# AD = AD, BC = BC, BD = BD, CD = CD, ABC = ABC, ABD = ABD,

# ACD = ACD, BCD = BCD, ABCD = ABCD)

## 5 group ##

# A <- setdiff(a, Reduce(union, list(b, c, d, e)))

# B <- setdiff(b, Reduce(union, list(a, c, d, e)))

# C <- setdiff(c, Reduce(union, list(a, b, d, e)))

# D <- setdiff(d, Reduce(union, list(a, b, c, e)))

# E <- setdiff(e, Reduce(union, list(a, b, c, d)))

#

# AB <- setdiff(intersect(a, b), Reduce(union, list(c, d, e)))

# AC <- setdiff(intersect(a, c), Reduce(union, list(b, d, e)))

# AD <- setdiff(intersect(a, d), Reduce(union, list(b, c, e)))

# AE <- setdiff(intersect(a, e), Reduce(union, list(b, c, d)))

# BC <- setdiff(intersect(b, c), Reduce(union, list(a, d, e)))

# BD <- setdiff(intersect(b, d), Reduce(union, list(a, c, e)))

# BE <- setdiff(intersect(b, e), Reduce(union, list(a, c, d)))

# CD <- setdiff(intersect(c, d), Reduce(union, list(a, b, e)))

# CE <- setdiff(intersect(c, e), Reduce(union, list(a, b, d)))

# DE <- setdiff(intersect(d, e), Reduce(union, list(a, b, c)))

#

# ABC <- setdiff(Reduce(intersect, list(a, b, c)), union(d, e))

# ABD <- setdiff(Reduce(intersect, list(a, b, d)), union(c, e))

# ABE <- setdiff(Reduce(intersect, list(a, b, e)), union(c, d))

# ACD <- setdiff(Reduce(intersect, list(a, c, d)), union(b, e))

# ACE <- setdiff(Reduce(intersect, list(a, c, e)), union(b, d))

# ADE <- setdiff(Reduce(intersect, list(a, d, e)), union(b, c))

# BCD <- setdiff(Reduce(intersect, list(b, c, d)), union(a, e))

# BCE <- setdiff(Reduce(intersect, list(b, c, e)), union(a, d))

# BDE <- setdiff(Reduce(intersect, list(b, d, e)), union(a, c))

# CDE <- setdiff(Reduce(intersect, list(c, d, e)), union(a, b))

#

# ABCD <- setdiff(Reduce(intersect, list(a, b, c, d)), e)

# ABCE <- setdiff(Reduce(intersect, list(a, b, c, e)), d)

# ABDE <- setdiff(Reduce(intersect, list(a, b, d, e)), c)

# ACDE <- setdiff(Reduce(intersect, list(a, c, d, e)), b)

# BCDE <- setdiff(Reduce(intersect, list(b, c, d, e)), a)

#

# ABCDE <- Reduce(intersect, list(a, b, c, d, e))

#

# items <- list(A = A, B = B, C = C, D = D, E = E,

# AB = AB, AC = AC, AD = AD, AE = AE,

# BC = BC, BD = BD, BE = BE,

# CD = CD, CE = CE, DE = DE,

# ABC = ABC, ABD = ABD, ABE = ABE, ACD = ACD, ACE = ACE, ADE = ADE,

# BCD = BCD, BCE = BCE, BDE = BDE, CDE = CDE,

# ABCD = ABCD, ABCE = ABCE, ABDE = ABDE,

# ACDE = ACDE, BCDE = BCDE,

# ABCDE = ABCDE)

library(VennDiagram)

venn.diagram(

x = items, filename = "veen.png",

imagetype="png" ,

height = 480,

width = 480,

resolution = 300

Scripts for volcano scripts

# library(tidyverse)

library(ggplot2)

data <- dat

data <- read.table("~/file.txt", header = T)

head(data)

# gene_name logFC pvalue padj

# 1 TSPAN6 -0.1847277 0.70495937 0.8875868

# 2 TNMD -0.5731570 0.66768318 0.8696220

# 3 DPM1 0.3804519 0.33123387 0.6587634

# 4 SCYL3 -0.6330844 0.22489833 0.5537215

# 5 C1orf112 0.0152763 0.98369157 0.9943630

# 6 FGR -1.0904422 0.01899727 0.1463301

data$col <- "no significant"

data$col[data$padj < 0.05 & data$logFC > 2] <- "Up"

data$col[data$padj < 0.05 & data$logFC < -2] <- "Down"

data$col <- factor(data$col, levels = c("Down", "no significant","Up"))

data$size <- 1

data$size[data$padj < 0.05 & data$logFC > 2] <- 2

data$size[data$padj < 0.05 & data$logFC < -2] <- 2

ggplot() +

geom_point(data = data, aes(logFC, -log10(padj), colour = col, fill = col),

size = data$size) +

scale_colour_manual(values = c("#4DBBD5", "grey", "#E64B35")) +

geom_vline(xintercept = c(-2, 2), color="grey40", linetype=2) +

geom_hline(yintercept = -log10(0.05), color="grey40", linetype=2)

Heatmap scripts

#### heatmap plotting 2017-10-19

####pheatmap example

library(pheatmap)

##creat a matrix

test = matrix(rnorm(200), 20, 10)

test[1:10, seq(1, 10, 2)] = test[1:10, seq(1, 10, 2)] + 3

test[11:20, seq(2, 10, 2)] = test[11:20, seq(2, 10, 2)] + 2

test[15:20, seq(2, 10, 2)] = test[15:20, seq(2, 10, 2)] + 4

colnames(test) = paste("Test", 1:10, sep = "")

rownames(test) = paste("Gene", 1:20, sep = "")

test

##Draw heatmap

pheatmap(test)

pheatmap(test, kmeans_k = 2)

pheatmap(test, scale = "column", clustering_distance_rows = "correlation")

pheatmap(test, scale = "row", clustering_distance_rows = "correlation")##Pearson correlation

pheatmap(test, color = colorRampPalette(c("navy", "white", "firebrick3"))(50))

pheatmap(test, cluster_row = FALSE)

pheatmap(test, legend = FALSE)

## Show text within cells

pheatmap(test, display_numbers = TRUE)##显示表达值

pheatmap(test, display_numbers = TRUE, number_format = "\%.1e")

pheatmap(test, display_numbers = matrix(ifelse(test > 5, "*", ""), nrow(test)))

pheatmap(test, cluster_row = FALSE, legend_breaks = -1:4, legend_labels = c("0",

"1e-4", "1e-3", "1e-2", "1e-1", "1"))

# Fix cell sizes and save to file with correct size

pheatmap(test, cellwidth = 15, cellheight = 12, main = "Example heatmap")##命名

pheatmap(test, cellwidth = 15, cellheight = 12, fontsize = 8, filename = "test.pdf")

# Generate annotations for rows and columns

annotation_col = data.frame(

CellType = factor(rep(c("CT1", "CT2"), 5)),

Time = 1:5

)

rownames(annotation_col) = paste("Test", 1:10, sep = "")

annotation_row = data.frame(

GeneClass = factor(rep(c("Path1", "Path2", "Path3"), c(10, 4, 6)))

)

rownames(annotation_row) = paste("Gene", 1:20, sep = "")

# Display row and color annotations

pheatmap(test, annotation_col = annotation_col)

pheatmap(test, annotation_col = annotation_col, annotation_legend = FALSE)

pheatmap(test, annotation_col = annotation_col, annotation_row = annotation_row)

# Specify colors

ann_colors = list(

Time = c("white", "firebrick"),

CellType = c(CT1 = "#1B9E77", CT2 = "#D95F02"),

GeneClass = c(Path1 = "#7570B3", Path2 = "#E7298A", Path3 = "#66A61E")

)

pheatmap(test, annotation_col = annotation_col, annotation_colors = ann_colors, main = "Title")

pheatmap(test, annotation_col = annotation_col, annotation_row = annotation_row,

annotation_colors = ann_colors)

pheatmap(test, annotation_col = annotation_col, annotation_colors = ann_colors[2])

# Gaps in heatmaps

pheatmap(test, annotation_col = annotation_col, cluster_rows = FALSE, gaps_row = c(10, 14))

pheatmap(test, annotation_col = annotation_col, cluster_rows = FALSE, gaps_row = c(10, 14),

cutree_col = 2)

# Show custom strings as row/col names

labels_row = c("", "", "", "", "", "", "", "", "", "", "", "", "", "", "",

"", "", "Il10", "Il15", "Il1b")

pheatmap(test, annotation_col = annotation_col, labels_row = labels_row)

##代码注释（转载于http://blog.sina.com.cn/s/blog_567b43d00101o546.html）

> library(pheatmap)

> pheatmap(data,fontsize=9, fontsize_row=6) #最简单地直接出图

> pheatmap(data, scale = "row", clustering_distance_row = "correlation", fontsize=9, fontsize_row=6) #改变排序算法

> pheatmap(data, color = colorRampPalette(c("navy", "white", "firebrick3"))(50), fontsize=9, fontsize_row=6) #自定义颜色

> pheatmap(data, cluster_row=FALSE, fontsize=9, fontsize_row=6) #关闭按行排序

> pheatmap(data, legend = FALSE, fontsize=9, fontsize_row=6) #关闭图例

> pheatmap(data, cellwidth = 6, cellheight = 5, fontsize=9, fontsize_row=6) #设定格子的尺寸

> color.map patientcolors hc dd.col groups annotation pheatmap(data, annotation=annotation, fontsize=9, fontsize_row=6) #为样品分组

> Var1 = c("navy", "skyblue")

> Var2 = c("snow", "steelblue")

> names(Var1) = c("class1", "class2")

> ann_colors = list(Var1 = Var1, Var2 = Var2)

> pheatmap(data, annotation=annotation, annotation_colors = ann_colors, fontsize=

Enrichment scripts

library(clusterProfiler)

library(org.Hs.eg.db) ## org.Mm.eg.db

gene_ids = bitr(geneID = gene_list, fromType = "SYMBOL",

toType = "ENTREZID", OrgDb = "org.Hs.eg.db")

head(gene_ids)

# SYMBOL ENTREZID

# 1 NAT1 9

# 2 ADH1B 125

# 3 BIRC5 332

# 4 AQP9 366

# 5 BCL2A1 597

# 6 BMP4 652

ego <- enrichGO(gene = gene_ids$ENTREZID, OrgDb = "org.Hs.eg.db",

keyType = "ENTREZID", ont = "BP")

# #

# # over-representation test

# #

# #...@organism Homo sapiens

# #...@ontology BP

# #...@keytype ENTREZID

# #...@gene chr [1:209] "9" "125" "332" "366" "597" "652" "730" "771" "776" "820" "890" "891" "983" "991" "1062" "1101" "1111" "1307" "1308" "1311" ...

# #...pvalues adjusted by 'BH' with cutoff <0.05

# #...187 enriched terms found

# 'data.frame': 187 obs. of 9 variables:

# $ ID : chr "GO:0140014" "GO:0000280" "GO:0048285" "GO:0000070" ...

# $ Description: chr "mitotic nuclear division" "nuclear division" "organelle fission" "mitotic sister chromatid segregation" ...

# $ GeneRatio : chr "32/196" "34/196" "35/196" "23/196" ...

# $ BgRatio : chr "264/18670" "407/18670" "449/18670" "151/18670" ...

# $ pvalue : num 7.53e-25 4.63e-21 1.10e-20 2.23e-20 2.61e-19 ...

# $ p.adjust : num 2.33e-21 7.16e-18 1.14e-17 1.72e-17 1.61e-16 ...

# $ qvalue : num 2.01e-21 6.16e-18 9.78e-18 1.48e-17 1.39e-16 ...

# $ geneID : chr "332/652/891/991/1062/1111/3832/3833/4085/4605/4751/6790/7272/9055/9212/9232/9319/9493/9787/10403/10460/11065/22"| __truncated__ "332/652/891/991/1062/1111/3832/3833/4085/4605/4751/6790/7153/7272/9055/9212/9232/9319/9493/9787/10403/10460/110"| __truncated__ "332/652/891/991/1062/1111/3832/3833/4085/4137/4605/4751/6790/7153/7272/9055/9212/9232/9319/9493/9787/10403/1046"| __truncated__ "891/991/1062/3833/4085/4751/7272/9055/9212/9232/9319/9493/9787/10403/10460/23397/24137/51203/55143/64151/81620/81930/146909" ...

# $ Count : int 32 34 35 23 24 28 20 24 17 19 ...

# #...Citation

# Guangchuang Yu, Li-Gen Wang, Yanyan Han and Qing-Yu He.

# clusterProfiler: an R package for comparing biological themes among

# gene clusters. OMICS: A Journal of Integrative Biology

# 2012, 16(5):284-287

#### enrichKEGG(gene = gene_ids$ENTREZID, organism = "hsa", keyType = "ENTREZID")

Enrichment graph scripts

library(clusterProfiler)

library(org.Hs.eg.db) ## org.Mm.eg.db

gene_ids = bitr(geneID = gene_list, fromType = "SYMBOL",

toType = "ENTREZID", OrgDb = "org.Hs.eg.db")

head(gene_ids)

# SYMBOL ENTREZID

# 1 NAT1 9

# 2 ADH1B 125

# 3 BIRC5 332

# 4 AQP9 366

# 5 BCL2A1 597

# 6 BMP4 652

ego <- enrichGO(gene = gene_ids$ENTREZID, OrgDb = "org.Hs.eg.db",

keyType = "ENTREZID", ont = "BP")

# #

# # over-representation test

# #

# #...@organism Homo sapiens

# #...@ontology BP

# #...@keytype ENTREZID

# #...@gene chr [1:209] "9" "125" "332" "366" "597" "652" "730" "771" "776" "820" "890" "891" "983" "991" "1062" "1101" "1111" "1307" "1308" "1311" ...

# #...pvalues adjusted by 'BH' with cutoff <0.05

# #...187 enriched terms found

# 'data.frame': 187 obs. of 9 variables:

# $ ID : chr "GO:0140014" "GO:0000280" "GO:0048285" "GO:0000070" ...

# $ Description: chr "mitotic nuclear division" "nuclear division" "organelle fission" "mitotic sister chromatid segregation" ...

# $ GeneRatio : chr "32/196" "34/196" "35/196" "23/196" ...

# $ BgRatio : chr "264/18670" "407/18670" "449/18670" "151/18670" ...

# $ pvalue : num 7.53e-25 4.63e-21 1.10e-20 2.23e-20 2.61e-19 ...

# $ p.adjust : num 2.33e-21 7.16e-18 1.14e-17 1.72e-17 1.61e-16 ...

# $ qvalue : num 2.01e-21 6.16e-18 9.78e-18 1.48e-17 1.39e-16 ...

# $ geneID : chr "332/652/891/991/1062/1111/3832/3833/4085/4605/4751/6790/7272/9055/9212/9232/9319/9493/9787/10403/10460/11065/22"| __truncated__ "332/652/891/991/1062/1111/3832/3833/4085/4605/4751/6790/7153/7272/9055/9212/9232/9319/9493/9787/10403/10460/110"| __truncated__ "332/652/891/991/1062/1111/3832/3833/4085/4137/4605/4751/6790/7153/7272/9055/9212/9232/9319/9493/9787/10403/1046"| __truncated__ "891/991/1062/3833/4085/4751/7272/9055/9212/9232/9319/9493/9787/10403/10460/23397/24137/51203/55143/64151/81620/81930/146909" ...

# $ Count : int 32 34 35 23 24 28 20 24 17 19 ...

# #...Citation

# Guangchuang Yu, Li-Gen Wang, Yanyan Han and Qing-Yu He.

# clusterProfiler: an R package for comparing biological themes among

# gene clusters. OMICS: A Journal of Integrative Biology

# 2012, 16(5):284-287

#### enrichKEGG(gene = gene_ids$ENTREZID, organism = "hsa", keyType = "ENTREZID")

### dot plot

dotplot(ego)

### cnet plot

cnetplot(ego)

library(clusterProfiler)

library(org.Hs.eg.db) ## org.Mm.eg.db

gene_ids = bitr(geneID = gene_list, fromType = "SYMBOL",

toType = "ENTREZID", OrgDb = "org.Hs.eg.db")

head(gene_ids)

# SYMBOL ENTREZID

# 1 NAT1 9

# 2 ADH1B 125

# 3 BIRC5 332

# 4 AQP9 366

# 5 BCL2A1 597

# 6 BMP4 652

ego <- enrichGO(gene = gene_ids$ENTREZID, OrgDb = "org.Hs.eg.db",

keyType = "ENTREZID", ont = "BP")

# #

# # over-representation test

# #

# #...@organism Homo sapiens

# #...@ontology BP

# #...@keytype ENTREZID

# #...@gene chr [1:209] "9" "125" "332" "366" "597" "652" "730" "771" "776" "820" "890" "891" "983" "991" "1062" "1101" "1111" "1307" "1308" "1311" ...

# #...pvalues adjusted by 'BH' with cutoff <0.05

# #...187 enriched terms found

# 'data.frame': 187 obs. of 9 variables:

# $ ID : chr "GO:0140014" "GO:0000280" "GO:0048285" "GO:0000070" ...

# $ Description: chr "mitotic nuclear division" "nuclear division" "organelle fission" "mitotic sister chromatid segregation" ...

# $ GeneRatio : chr "32/196" "34/196" "35/196" "23/196" ...

# $ BgRatio : chr "264/18670" "407/18670" "449/18670" "151/18670" ...

# $ pvalue : num 7.53e-25 4.63e-21 1.10e-20 2.23e-20 2.61e-19 ...

# $ p.adjust : num 2.33e-21 7.16e-18 1.14e-17 1.72e-17 1.61e-16 ...

# $ qvalue : num 2.01e-21 6.16e-18 9.78e-18 1.48e-17 1.39e-16 ...

# $ geneID : chr "332/652/891/991/1062/1111/3832/3833/4085/4605/4751/6790/7272/9055/9212/9232/9319/9493/9787/10403/10460/11065/22"| __truncated__ "332/652/891/991/1062/1111/3832/3833/4085/4605/4751/6790/7153/7272/9055/9212/9232/9319/9493/9787/10403/10460/110"| __truncated__ "332/652/891/991/1062/1111/3832/3833/4085/4137/4605/4751/6790/7153/7272/9055/9212/9232/9319/9493/9787/10403/1046"| __truncated__ "891/991/1062/3833/4085/4751/7272/9055/9212/9232/9319/9493/9787/10403/10460/23397/24137/51203/55143/64151/81620/81930/146909" ...

# $ Count : int 32 34 35 23 24 28 20 24 17 19 ...

# #...Citation

# Guangchuang Yu, Li-Gen Wang, Yanyan Han and Qing-Yu He.

# clusterProfiler: an R package for comparing biological themes among

# gene clusters. OMICS: A Journal of Integrative Biology

# 2012, 16(5):284-287

#### enrichKEGG(gene = gene_ids$ENTREZID, organism = "hsa", keyType = "ENTREZID")

### dot plot

dotplot(ego)

### cnet plot

cnetplot(ego)

scripts for survive analysis

if (!requireNamespace("survminer", quietly = TRUE))

install.packages("survminer")

library(survival)

library(survminer)

# data <- lung

# colnames(data)[5] <- "variable"

fit <- survfit(Surv(time, status) ~ variable, data = data)

print(fit)

# Call: survfit(formula = Surv(time, status) ~ variable, data = data)

#

# n events median 0.95LCL 0.95UCL

# variable=1 138 112 270 212 310

# variable=2 90 53 426 348 550

survdiff(Surv(time, status) ~ variable, data = data)

# survdiff(formula = Surv(time, status) ~ variable, data = data)

#

# N Observed Expected (O-E)^2/E (O-E)^2/V

# variable=1 138 112 91.6 4.55 10.3

# variable=2 90 53 73.4 5.68 10.3

#

# Chisq= 10.3 on 1 degrees of freedom, p= 0.001

fit2 <- coxph(Surv(time, status) ~ variable, data = data)

summary(fit2)

# Call:

# coxph(formula = Surv(time, status) ~ variable, data = data)

#

# n= 228, number of events= 165

#

# coef exp(coef) se(coef) z Pr(>|z|)

# variable -0.5310 0.5880 0.1672 -3.176 0.00149 **

# ---

# Signif. codes: 0 ‘***’ 0.001 ‘**’ 0.01 ‘*’ 0.05 ‘.’ 0.1 ‘ ’ 1

#

# exp(coef) exp(-coef) lower .95 upper .95

# variable 0.588 1.701 0.4237 0.816

#

# Concordance= 0.579 (se = 0.021 )

# Likelihood ratio test= 10.63 on 1 df, p=0.001

# Wald test = 10.09 on 1 df, p=0.001

# Score (logrank) test = 10.33 on 1 df, p=0.001

# plot

ggsurvplot(fit = fit, data = data, pval = T)

Scripts for immune infiltration

# library(tidyverse)

library(GSVA)

library(clusterProfiler)

library(org.Hs.eg.db)

library(data.table)

library(rtracklayer)

### ssGSEA ######

## table S1 - https://doi.org/10.1016/j.immuni.2013.10.003

## pdf -> table -> read

immunity <- read.csv("~/immunity-cell-gene.csv", header = T)

# CellType AffymetrixID Symbol Gene.Symbol ENTREZ_GENE_ID

# 1 aDC 205569_at LAMP3 LAMP3 27074

# 2 aDC 207533_at CCL1 CCL1 6346

# 3 aDC 210029_at INDO IDO1 3620

# 4 aDC 218400_at OAS3 OAS3 4940

# 5 aDC 219424_at EBI3 EBI3 10148

# 6 B cells 204836_at GLDC GLDC 2731

idx <- !immunity$CellType %in% c("Blood vessels", "Normal mucosa", "SW480 cancer cells", "Lymph vessels")

immunity <- immunity[idx,]

immunity <- immunity %>%

split(., .$CellType) %>%

lapply(., function(x)(x$ENTREZ_GENE_ID))

immunity <- lapply(immunity, unique)

## Ensembl download

anno <- import('~/Homo_sapiens.GRCh38.101.gtf')

anno <- as.data.frame(anno)

anno <- anno[!duplicated(anno$gene_id),]

anno <- merge(anno, gene_symbol, by = "gene_name")

anno <- rbind(anno, data.frame(gene_name = c("KIAA1324", "IGHA1"),

gene_id = c("ENSG00000116299", "ENSG00000211895"),

ENTREZID = c("57535", "3492")))

anno <- anno[!duplicated(anno$gene_id),] ### 37417

anno <- anno[, c("gene_id", "ENTREZID")]

data <- fread("~/tpm.txt") %>%

rename("gene_id" = "V1") %>%

left_join(., anno, by = "gene_id") %>%

filter(!is.na(ENTREZID)) %>%

select(-gene_id) %>%

column_to_rownames("ENTREZID")

data <- log2(data + 1)

immu_cell <- as.data.frame(gsva(as.matrix(data), immunity, method = "ssgsea"))

### bbt plot

data <- read.table("~/file.txt", header = T)

# group aDC B cells CD8 T cells Cytotoxic cells

# 1 1.13092315 0.4709550 0.26202395 0.5944611 0.5130117

# 3 0.55644003 0.1800251 -0.07081909 0.5197230 0.2135559

# 4 0.44696904 0.3350859 0.05579749 0.5908900 0.3135561

# 5 0.05474605 0.1191767 0.02578815 0.5541712 0.2068595

# 7 0.61364297 0.1563856 0.09869185 0.5518254 0.2321028

# 8 0.41079217 0.4588979 0.50105493 0.5996277 0.4866508

data1 <- NULL

for(i in 2:25){

cor <- cor.test(data[,i], data[,1], method = "pearson")

data1 <- rbind(data1,

data.frame("group" = "a",

"cell" = colnames(data)[i],

"cor" = cor$estimate,

"p" = cor$p.value))

}

data1 <- data1[order(data1$cor),]

data1$cell <- factor(data1$cell, levels = data1$cell)

ggplot(data1, aes(x = cell, y = cor)) +

geom_segment(aes(xend=cell,yend=0)) +

geom_hline(yintercept = 0) +

geom_point(aes(col=p, fill = p, size=abs(cor))) +

coord_flip()

# library(tidyverse)

library(GSVA)

library(clusterProfiler)

library(org.Hs.eg.db)

library(data.table)

library(rtracklayer)

### ssGSEA ######

## table S1 - https://doi.org/10.1016/j.immuni.2013.10.003

## pdf -> table -> read

immunity <- read.csv("~/immunity-cell-gene.csv", header = T)

# CellType AffymetrixID Symbol Gene.Symbol ENTREZ_GENE_ID

# 1 aDC 205569_at LAMP3 LAMP3 27074

# 2 aDC 207533_at CCL1 CCL1 6346

# 3 aDC 210029_at INDO IDO1 3620

# 4 aDC 218400_at OAS3 OAS3 4940

# 5 aDC 219424_at EBI3 EBI3 10148

# 6 B cells 204836_at GLDC GLDC 2731

idx <- !immunity$CellType %in% c("Blood vessels", "Normal mucosa", "SW480 cancer cells", "Lymph vessels")

immunity <- immunity[idx,]

immunity <- immunity %>%

split(., .$CellType) %>%

lapply(., function(x)(x$ENTREZ_GENE_ID))

immunity <- lapply(immunity, unique)

## Ensembl download

anno <- import('~/Homo_sapiens.GRCh38.101.gtf')

anno <- as.data.frame(anno)

anno <- anno[!duplicated(anno$gene_id),]

anno <- merge(anno, gene_symbol, by = "gene_name")

anno <- rbind(anno, data.frame(gene_name = c("KIAA1324", "IGHA1"),

gene_id = c("ENSG00000116299", "ENSG00000211895"),

ENTREZID = c("57535", "3492")))

anno <- anno[!duplicated(anno$gene_id),] ### 37417

anno <- anno[, c("gene_id", "ENTREZID")]

data <- fread("~/tpm.txt") %>%

rename("gene_id" = "V1") %>%

left_join(., anno, by = "gene_id") %>%

filter(!is.na(ENTREZID)) %>%

select(-gene_id) %>%

column_to_rownames("ENTREZID")

data <- log2(data + 1)

immu_cell <- as.data.frame(gsva(as.matrix(data), immunity, method = "ssgsea"))

### group plot

data <- read.table("~/file.txt", header = T)

# group aDC

# 1 1.13092315 0.4709550

# 3 0.55644003 0.1800251

# 4 0.44696904 0.3350859

# 5 0.05474605 0.1191767

# 7 0.61364297 0.1563856

# 8 0.41079217 0.4588979

colnames(data) <- c("x", "y")

data$group <- ifelse(data$x >= median(data$x), "High", "Low")

data$group <- factor(data$group, levels = c("Low", "High"))

ggplot(data, aes(x = group, y = y, color = group, fill = group)) +

geom_boxplot(alpha = 0.2) +

geom_point(position = position_jitter(0.3)) +

theme_bw()

# library(tidyverse)

library(GSVA)

library(clusterProfiler)

library(org.Hs.eg.db)

library(data.table)

library(rtracklayer)

### ssGSEA ######

## table S1 - https://doi.org/10.1016/j.immuni.2013.10.003

## pdf -> table -> read

immunity <- read.csv("~/immunity-cell-gene.csv", header = T)

# CellType AffymetrixID Symbol Gene.Symbol ENTREZ_GENE_ID

# 1 aDC 205569_at LAMP3 LAMP3 27074

# 2 aDC 207533_at CCL1 CCL1 6346

# 3 aDC 210029_at INDO IDO1 3620

# 4 aDC 218400_at OAS3 OAS3 4940

# 5 aDC 219424_at EBI3 EBI3 10148

# 6 B cells 204836_at GLDC GLDC 2731

idx <- !immunity$CellType %in% c("Blood vessels", "Normal mucosa", "SW480 cancer cells", "Lymph vessels")

immunity <- immunity[idx,]

immunity <- immunity %>%

split(., .$CellType) %>%

lapply(., function(x)(x$ENTREZ_GENE_ID))

immunity <- lapply(immunity, unique)

## Ensembl download

anno <- import('~/Homo_sapiens.GRCh38.101.gtf')

anno <- as.data.frame(anno)

anno <- anno[!duplicated(anno$gene_id),]

anno <- merge(anno, gene_symbol, by = "gene_name")

anno <- rbind(anno, data.frame(gene_name = c("KIAA1324", "IGHA1"),

gene_id = c("ENSG00000116299", "ENSG00000211895"),

ENTREZID = c("57535", "3492")))

anno <- anno[!duplicated(anno$gene_id),] ### 37417

anno <- anno[, c("gene_id", "ENTREZID")]

data <- fread("~/tpm.txt") %>%

rename("gene_id" = "V1") %>%

left_join(., anno, by = "gene_id") %>%

filter(!is.na(ENTREZID)) %>%

select(-gene_id) %>%

column_to_rownames("ENTREZID")

data <- log2(data + 1)

immu_cell <- as.data.frame(gsva(as.matrix(data), immunity, method = "ssgsea"))

### cor plot

data <- read.table("~/file.txt", header = T)

# group aDC

# 1 1.13092315 0.4709550

# 3 0.55644003 0.1800251

# 4 0.44696904 0.3350859

# 5 0.05474605 0.1191767

# 7 0.61364297 0.1563856

# 8 0.41079217 0.4588979

colnames(data) <- c("x", "y")

ggplot(data, aes(x = x, y = y)) +

geom_point() +

geom_smooth(formula = y ~ x, method = "lm") +

theme_bw()

Scripts for correlation analysis for two genes

library(ggplot2)

set.seed(100)

data <- data.frame(x = rnorm(100, 2, 1), y = rnorm(100, 1, 1))

data2 <- melt(data)

data3 <- lapply(data, function(x) get_summary_stats(data.frame(x)))

data3

# $x

# # A tibble: 1 x 13

# variable n min max median q1 q3 iqr mad mean sd se ci

# <chr> <dbl> <dbl> <dbl> <dbl> <dbl> <dbl> <dbl> <dbl> <dbl> <dbl> <dbl> <dbl>

# 1 x 100 -0.272 4.58 1.94 1.39 2.66 1.26 0.974 2.00 1.02 0.102 0.203

#

# $y

# # A tibble: 1 x 13

# variable n min max median q1 q3 iqr mad mean sd se ci

# <chr> <dbl> <dbl> <dbl> <dbl> <dbl> <dbl> <dbl> <dbl> <dbl> <dbl> <dbl> <dbl>

# 1 x 100 -1.14 3.17 0.927 0.568 1.45 0.878 0.648 1.01 0.796 0.08 0.158

cor.test(data[,1], data[,2], method = "pearson")

# Pearson's product-moment correlation

#

# data: data[, 1] and data[, 2]

# t = -1.1205, df = 98, p-value = 0.2652

# alternative hypothesis: true correlation is not equal to 0

# 95 percent confidence interval:

# -0.30221314 0.08584318

# sample estimates:

# cor

# -0.1124713

cor.test(data[,1], data[,2], method = "spearman")

# Spearman's rank correlation rho

#

# data: data[, 1] and data[, 2]

# S = 192064, p-value = 0.1297

# alternative hypothesis: true rho is not equal to 0

# sample estimates:

# rho

# -0.1524992

ggplot(data, aes(x = x, y = y)) +

geom_point() +

geom_smooth(formula = y ~ x, method = "lm") +

theme_bw()
