# Supplementary material for: Exercise-induced IL-15 acted as a positive prognostic implication and tumor-suppressed role in pan-cancer
Source: Front Pharmacol. 2022 Nov 17;13:1053137. doi: 10.3389/fphar.2022.1053137 (PMC9712805; doi:10.3389/fphar.2022.1053137)
Supplement: Supplementary file 3 [file DataSheet1.docx]

Supplementary Material

# Supplementary Figures and Tables

## Supplementary Figures


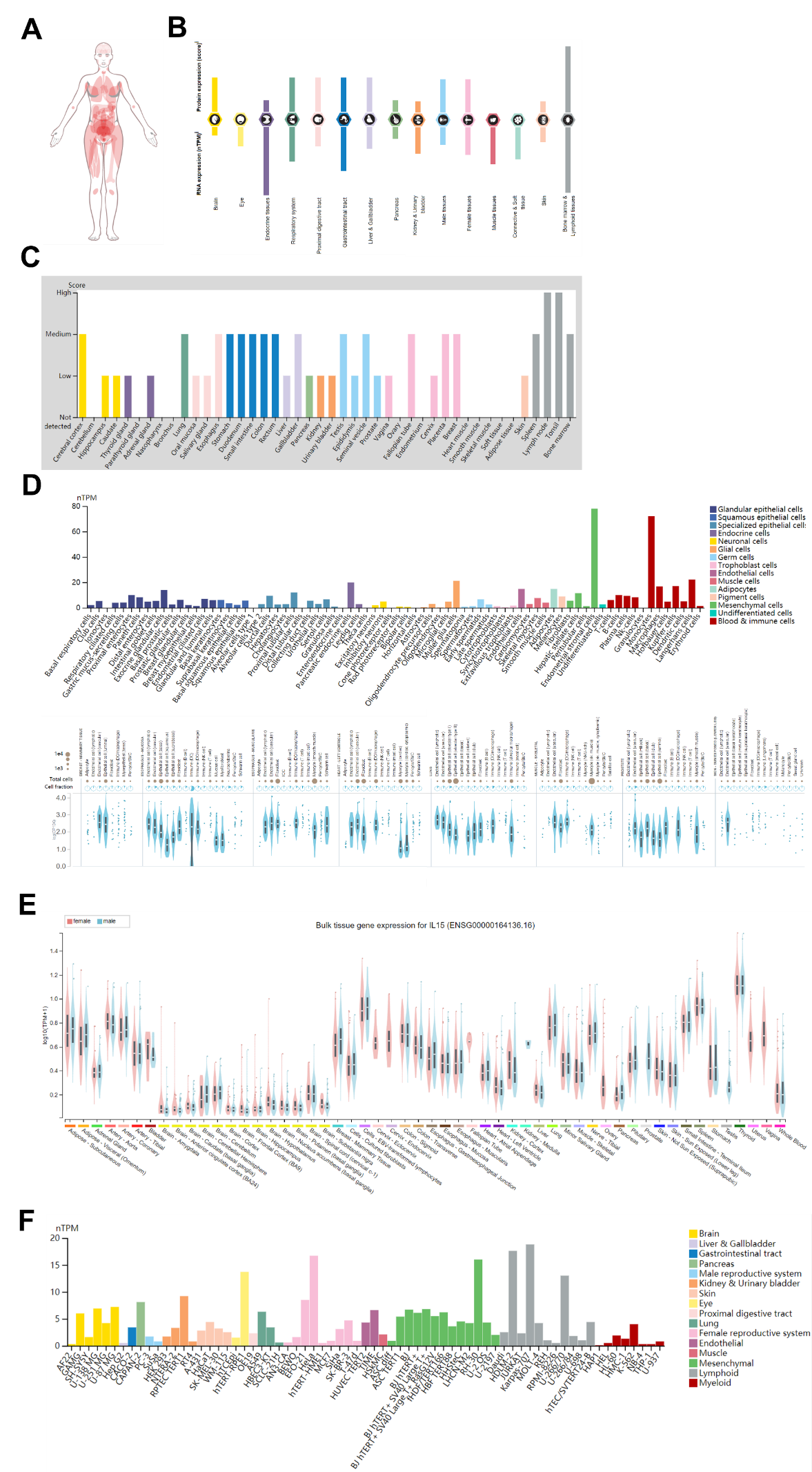


**Supplementary Figure 1.** Differential expression pattern of IL-15. Related to Figure 1. **(A-D)** The expression distribution diagram for different organs **(A-B)**, tissue **(C)**, and cell types **(D)**. **(E)** Gender comparison for IL-15 expression in different organs. **(F)** IL-15 expression levels in different cancer cell lines.


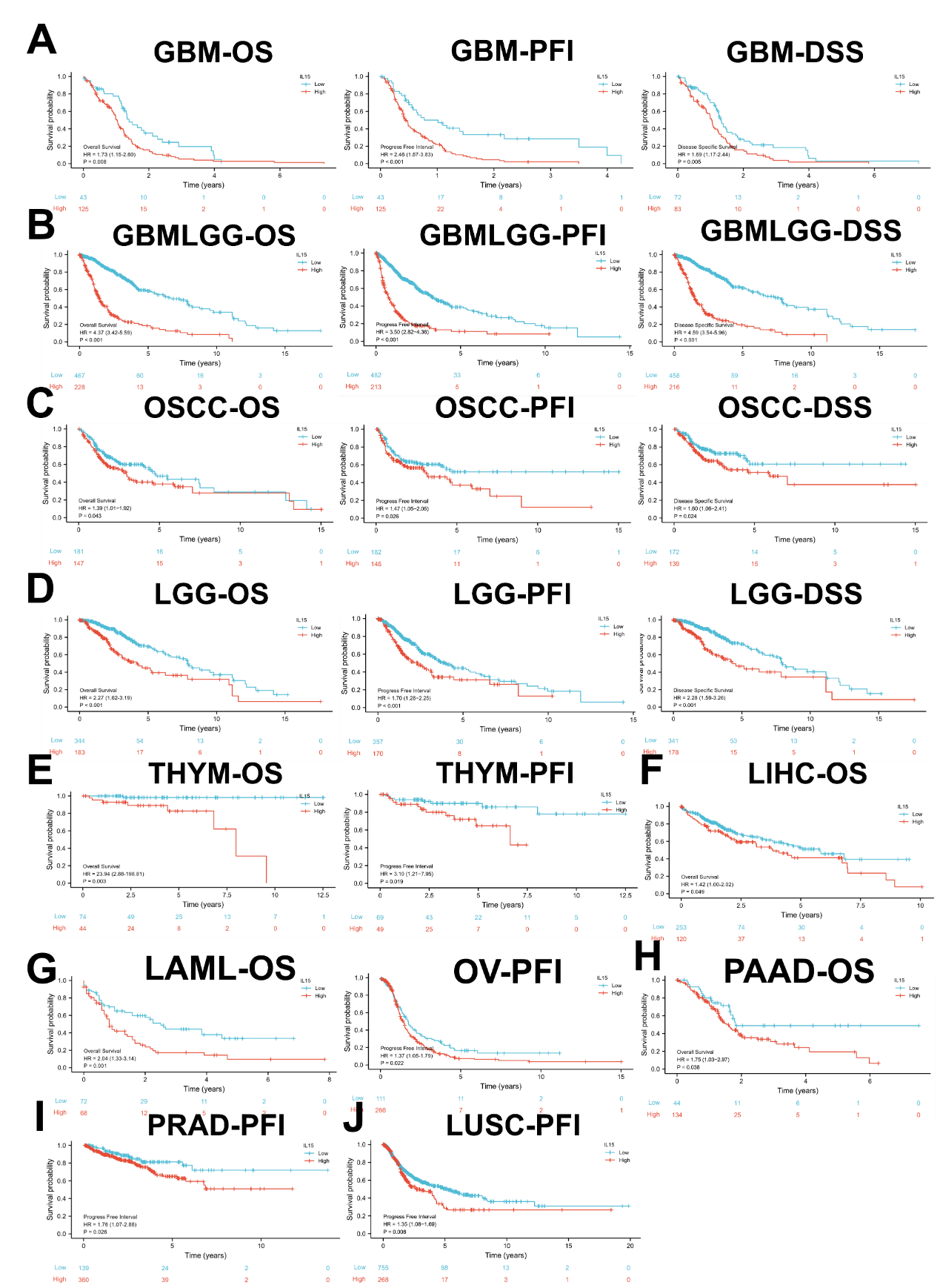


**Supplementary Figure 2.** Patient survival period is based on the different expressions of IL-15. **(A-J)** Kaplan–Meier analysis of the association between IL-15 expression and OS/DSS/PFI in GBM, GBMLGG, OSCC, LGG, THYM LAML, and PRAD. The red line shows high IL-15 expression, and the blue line represents low IL-15 expression. OS, overall survival; DSS, disease-specific survival; PFI: progression-free interval.


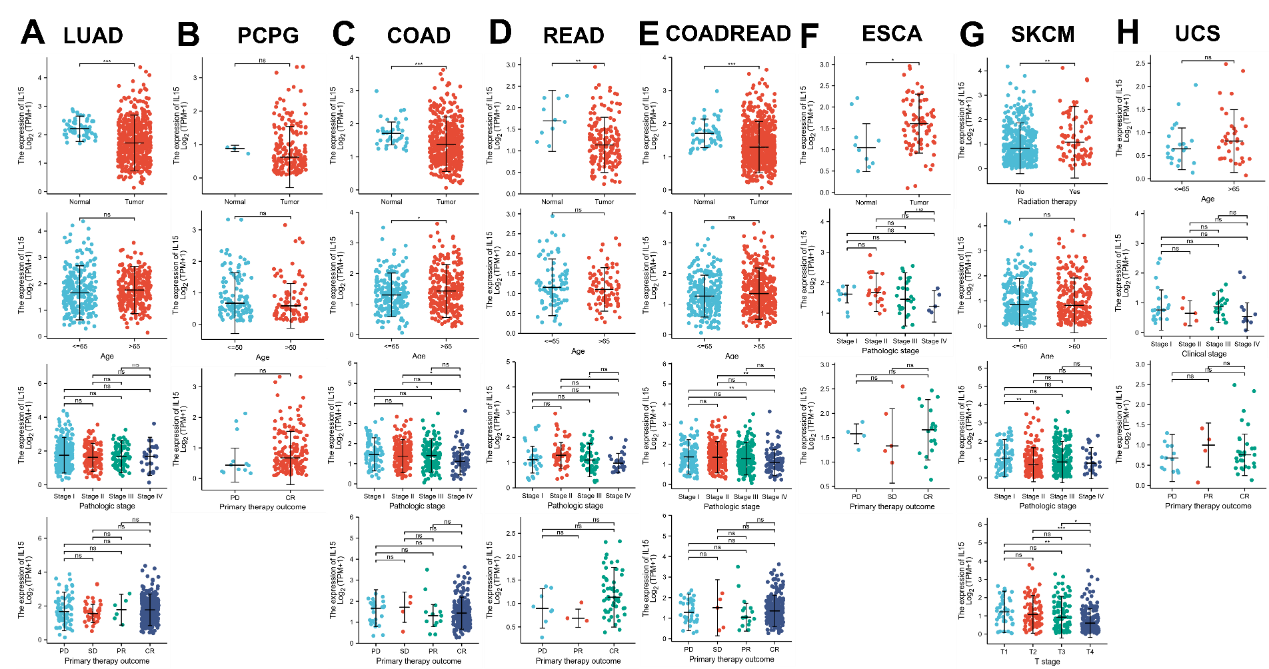


**Supplementary Figure 3.** Correlation between the mRNA expression level of IL-15 and patients’ clinicopathological features progression in pan-cancer. **(A-H)** Age, tumor stage, tumor-free or not, and tumor treatment response were compared for IL-15 expression in LUAD **(A)**, PCPG **(B)**, COAD **(C)**, READ **(D)**, COADREAD **(E)**, ESCA **(F)**, SKCM **(G)**, and UCS **(H)**. *p<0.05, **p <0.01, and ***p<0.001.


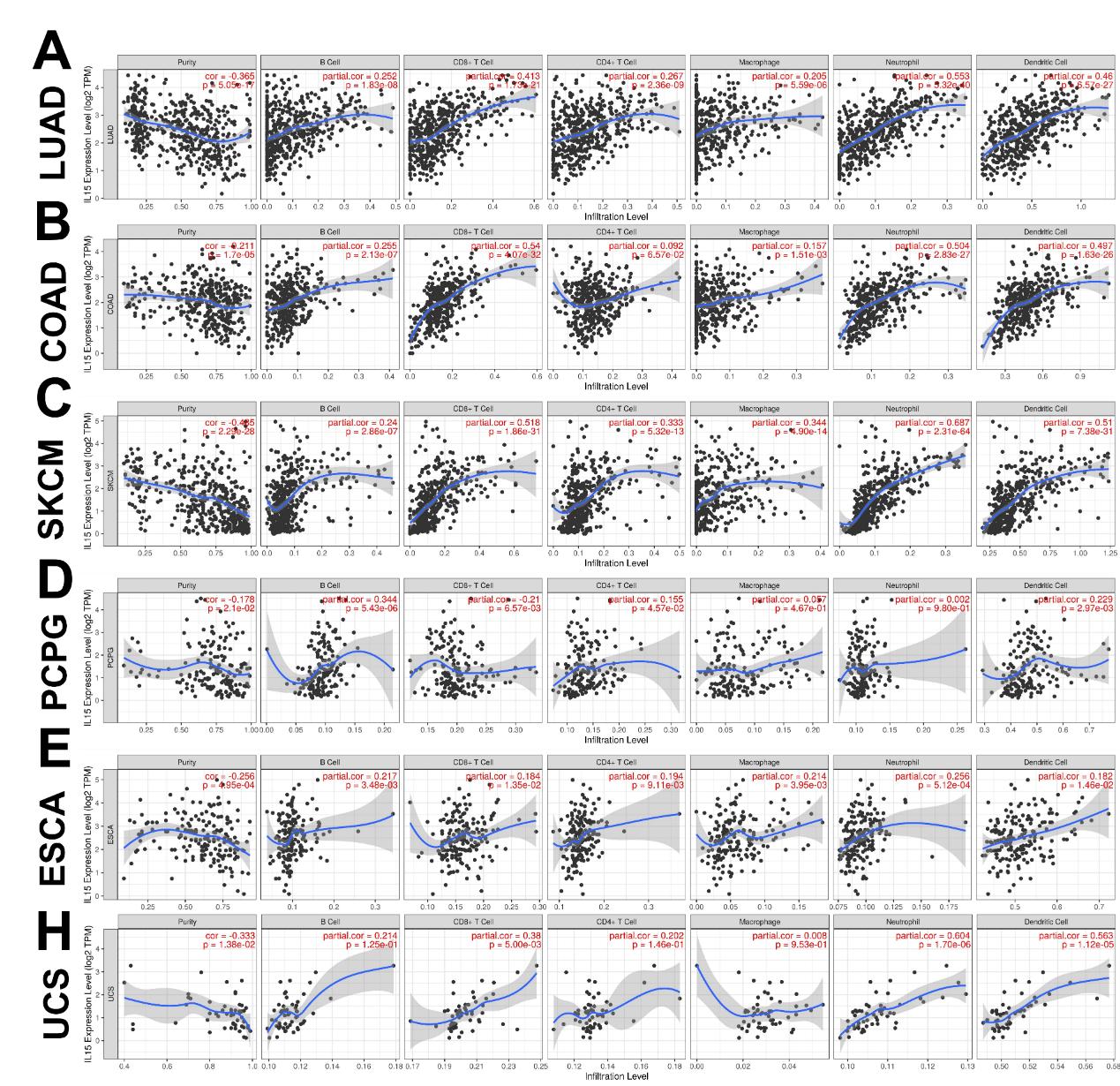


**Supplementary Figure 4.** Correlation between IL-15 gene expression and tumor immune microenvironment in TCGA database. **(A–H)** Analysis of immune-associated cells (B cells, CD8+ T cells, Macrophages, Neutrophils, Dendritic cells) infiltration with IL-15 expression in LUAD **(A)**, PCPG **(B)**, COAD **(C)**, READ **(D)**, COADREAD **(E)**, ESCA **(F)**, SKCM **(G)**, and UCS **(H)**.
